# Validation of a 2-Gene Blood Test for Kawasaki Disease in Febrile Children

**DOI:** 10.1001/jamanetworkopen.2026.15420

**Published:** 2026-05-28

**Authors:** Ho-Chang Kuo, Xing Xue, Fang Liu, Richard D. Mortensen, C. James Chou, Bo Jin, Juan Wei, Qiong Luo, Ken-Pen Weng, Mindy Ming-Huey Guo, Kuender D. Yang, Kuo-Jung Su, Shih-Ting Kang, Sun Kim, Weiwei Li, James Schilling, Zhi Han, Naoto Ozawa, Takumi Ichikawa, Henry Chubb, Scott R. Ceresnak, Gary L. Darmstadt, Doff McElhinney, Harvey J. Cohen, Xuefeng B. Ling

**Affiliations:** 1 College of Medicine Chang Gung University, Taoyuan, Taiwan; 2 Kawasaki Disease Center and Department of Pediatrics, Kaohsiung Chang Gung Memorial Hospital and Chang Gung University College of Medicine, Kaohsiung, Taiwan; 3 Department of Respiratory Therapy, Kaohsiung Chang Gung Memorial Hospital, Kaohsiung, Taiwan; 4 Heart Center, Children’s Hospital of Fudan University, Shanghai, China; 5 OncoOmicsDx Clinical Laboratory, Rockville, Maryland; 6 Women’s Hospital, Zhejiang University School of Medicine, Hangzhou, China; 7 Congenital Structural Heart Disease Center, Department of Pediatrics Kaohsiung Veterans General Hospital, Kaohsiung, Taiwan; 8 MacKay Children’s Hospital, Taipei, Taiwan; 9 School of Medicine, Stanford University, Stanford, California; 10 Nippon Life Insurance Company, Osaka, Japan; 11 Institute for Translational Research in Biomedicine, Kaohsiung Chang Gung Memorial Hospital and Chang Gung University College of Medicine, Kaohsiung, Taiwan

## Abstract

**Importance:**

Kawasaki disease (KD) remains a clinical diagnosis without an objective molecular test. Early identification is critical to prevent coronary artery complications through timely intravenous immunoglobulin therapy.

**Objective:**

To validate a 2-gene whole-blood quantitative polymerase chain reaction (qPCR) assay measuring *IFI27* and *MCEMP1* expression for distinguishing KD from other pediatric febrile illnesses.

**Design, Setting, and Participants:**

This multicenter diagnostic study was conducted in Taiwan and Shanghai, China. Patient blood samples were collected prospectively between 2012 and 2023 in Taiwan and between 2022 and 2023 in Shanghai and analyzed retrospectively from children younger than 8 years with KD and febrile controls (FCs) with viral, bacterial, or mixed infections. Data were analyzed from January 2022 to August 2025.

**Main Outcomes and Measures:**

Diagnostic accuracy of a prespecified 2-gene KD score derived from change in cycle threshold values normalized to glyceraldehyde 3-phosphate dehydrogenase, assessed by area under the receiver operating characteristic curve (AUC), sensitivity, specificity, predictive values, and likelihood ratios.

**Results:**

A total of 541 children (mean [SD] age, 3.7 [1.9] years; 300 [55.5%] male), including 243 children with KD and 298 febrile controls, were analyzed. The KD score achieved an AUC of 0.91 (95% CI, 0.88-0.94), with a sensitivity of 94% (95% CI, 93%-97%) and a specificity of 82% (95% CI, 78%-86%). The positive likelihood ratio was 5.12, and the negative likelihood ratio was 0.05. Performance was consistent across cohorts, including incomplete KD, diverse FC etiologies, and coronary artery phenotypes. The assay was implemented as a laboratory-developed test. Analytical validation demonstrated high linearity (*R*^2^ > 0.99), precision (coefficient of variation <5%), and sample stability for up to 6 days at 4 °C or for 24 hours at room temperature.

**Conclusions and Relevance:**

This diagnostic study found that a 2-gene laboratory-developed whole-blood qPCR assay measuring *IFI27* and *MCEMP1* expression accurately distinguished KD from other febrile illnesses using standard molecular platforms. Prospective evaluation in broader populations is warranted to determine its clinical utility for reducing diagnostic delay and coronary complications.

## Key Points

**Question:**

Can a 2-gene blood test distinguish Kawasaki disease from other febrile illnesses in children?

**Findings:**

In this diagnostic study of 541 febrile children, expression of *IFI27* and *MCEMP1* measured by quantitative polymerase chain reaction accurately identified Kawasaki disease. Performance was consistent across 5 independent cohorts, including incomplete Kawasaki disease, diverse febrile-control etiologies, and coronary artery phenotypes.

**Meaning:**

These findings suggest that a simple 2-gene blood test may provide an objective adjunct to clinical criteria for early Kawasaki disease diagnosis and reduce delays in initiating intervention.

## Teaser

This diagnostic study validates a whole-blood quantitative polymerase chain reaction assay measuring *IFI27* and *MCEMP1* expression for distinguishing Kawasaki disease from other pediatric febrile illnesses.

## Introduction

Kawasaki disease (KD) is an acute vasculitis of childhood and the leading cause of acquired heart disease in developed countries.^1,2,3,4^ Timely treatment with intravenous immunoglobulin reduces coronary complications,^5,6,7,8^ yet diagnosis remains clinical and subjective.^9^ Up to 25% of children present with incomplete features overlapping viral or bacterial infections, leading to diagnostic delay.

Prior studies^9,10,11,12,13,14^ have explored molecular biomarkers to distinguish KD from other febrile illnesses. Transcriptomic investigations have reported multigene classifiers,^15,16,17,18^ but most remain research tools, limited by assay complexity and lack of independent clinical validation.

A 2-gene whole-blood signature of *IFI27* and *MCEMP1* was previously identified through integrated transcriptomic analyses of public datasets (eFigure 1, eTables 1-4, and eMethods in Supplement 1). To determine its clinical utility, we evaluated this signature using a quantitative polymerase chain reaction (qPCR) assay across 2 medical center cohorts in Taiwan and Shanghai to assess diagnostic accuracy, reproducibility, and analytical robustness under blinded conditions.

## Methods

This diagnostic study was approved by institutional review boards at all participating centers, and written informed consent was obtained from a parent or guardian for all participants. Molecular testing and clinical adjudication were performed independently under blinded conditions in accordance with Standards for Reporting of Diagnostic Accuracy (STARD) reporting guideline.

### Study Design and Setting

**Figure 1.**
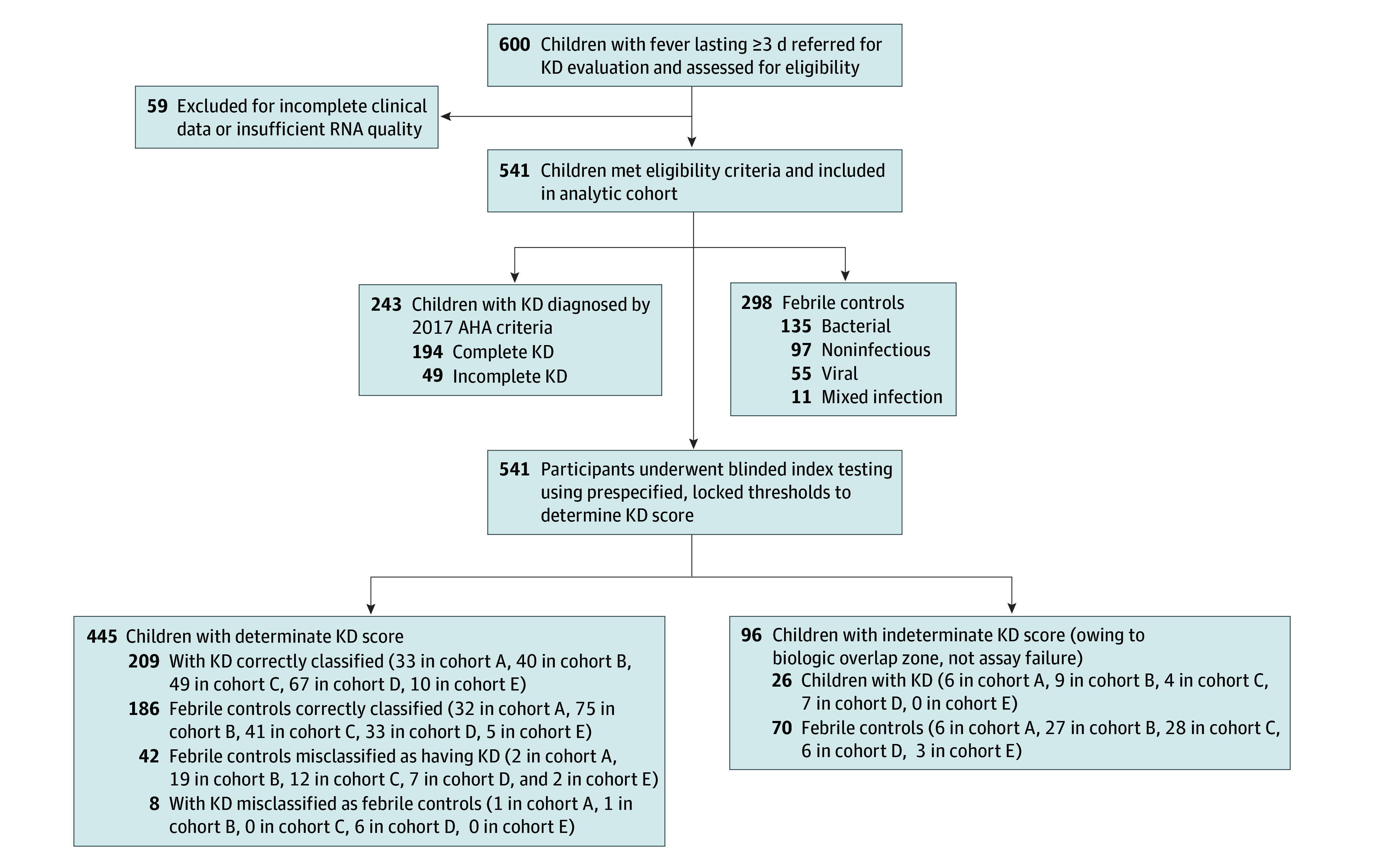
Participant Enrollment Flowchart of the *IFI27*-*MCEMP1* Diagnostic Test Of 600 febrile children assessed, 59 were excluded, leaving 541 participants (243 with Kawasaki disease [KD] and 298 febrile controls) who underwent blinded *IFI27*-*MCEMP1* quantitative polymerase chain reaction testing using locked thresholds. Determinate classifications were obtained in 445 participants (209 patients with KD correctly classified; 8 patients with KD misclassified; 186 febrile controls correctly classified; 42 febrile controls misclassified). Ninety-six participants (26 KD, 70 FC) fell within the prespecified indeterminate range, reflecting biologic overlap rather than assay failure. KD diagnoses were adjudicated by 2 pediatric cardiologists using 2017 American Heart Association (AHA) criteria.

This multicenter diagnostic accuracy study prospectively enrolled children (Figure 1) with suspected KD between June 2012 and March 2023 in Taiwan and November 2022 and June 2023 in Shanghai, China, at tertiary referral hospitals. Peripheral blood samples were obtained at initial evaluation.

### Participants

Children younger than age 8 years presenting with fever for at least 3 days and clinical suspicion of KD were prospectively screened at the participating hospitals during the study period (Table 1; eTable 5 in Supplement 1). Children with KD were defined according to the 2017 American Heart Association (AHA) diagnostic criteria for complete or incomplete KD.^19^ Patients meeting these criteria based on clinical features, laboratory findings, and echocardiographic evaluation were classified as having KD.

**Table 1.** Demographic and Clinical Characteristics and Laboratory Test Results for Patients With KD and FCs in the 5 Validation Cohorts^a^

Characteristics	Cohort A^b^			Cohort B^b^			Cohort C^c^			Cohort D^c^			Cohort E^d^		
	Patients, No. (%)		*P* value	Patients, No. (%)		*P* value	Patients, No. (%)		*P* value	Patients, No. (%)		*P* value	Patients, No. (%)		*P* value
	FC	KD		FC	KD		FC	KD		FC	KD		FC	KD	
Patients, No.	40	40	NA	121	60	NA	81	53	NA	46	80	NA	10	10	NA
Sex															
Female	19 (47.5)	18 (45.0)	.74	50 (41.3)	25 (41.7)	.92	40 (49.4)	23 (43.4)	.50	20 (43.5)	32 (40.0)	.70	5 (50.0)	7 (70.0)	.65
Male sex	20 (50.0)	22 (55.0)		69 (57.0)	35 (58.3)		41 (50.6)	30 (56.6)		26 (56.5)	48 (60.0)		5 (50.0)	3 (30.0)	
Age, median (IQR), y	2.7 (1.6-4.1)	1.2 (0.8-2.5)	.001	4.2 (2.5-5.5)	1.4 (0.9-2.4)	<.001	3.4 (1.7-5.5)	2.7 (1.3-4.2)	.03	2 (1.4-3.6)	1.9 (1.1-3.4)	.25	6.67 (4.05-7.29)	2.08 (1.21-3.46)	.03
Febrile day at sample collection, median (IQR)	5 (4-5)	7 (6-8)	<.001	4 (3-5)	7 (6-8)	<.001	1 (1-3)	6 (5-7)	<.001	1 (1-3)	5.5 (4-6)	<.001	5 (4-7)	6 (5-6)	.39
Principal KD symptoms^e^															
Lip/oral	1 (2.5)	38 (95.0)	.99	2 (1.7)	54 (90.0)	.99	2 (2.5)	53 (100)	<.001	0	74 (92.5)	<.001	0	9 (90.0)	NA
Conjunctival	3 (7.5)	38 (95.0)	.99	7 (5.8)	58 (96.7)	.99	1 (1.2)	53 (100)	<.001	0	74 (92.5)	<.001	1 (10)	8 (80)	.99
Cervical LN	13 (32.5)	11 (27.5)	<.001	12 (9.9)	25 (41.7)	<.001	2 (2.5)	38 (71.7)	<.001	0	50 (62.5)	<.001	3 (30)	3 (30.0)	.07
Extremity	1 (2.5)	30 (75.0)	.99	1 (0.8)	59 (98.3)	.99	1 (1.2)	34 (64.2)	<.001	0	64 (80.0)	<.001	0	2 (20.0)	NA
Rash	5 (12.5)	38 (95.0)	.99	12 (9.9)	57 (95.0)	.99	6 (7.4)	43 (81.1)	<.001	1 (2.2)	62 (77.5)	<.001	4 (40.0)	10 (100)	NA
Laboratory values, median (IQR)															
Hemoglobin, g/dL	11.9 (11.2-12.6)	10.6 (9.7-11.7)	<.001	12.2 (11.6-12.7)	11.1 (10.6-11.6)	<.001	12.1 (11.6-12.9)	10.9 (10.4-11.5)	<.001	12.3 (0.9)	10.9 (1.1)	<.001	11.3 (10.9-12.2)	11.2 (10.5-12.2)	.55
CRP, mg/dL	0.73 (0.23-4.69)	6.02 (2.90-10.67)	<.001	2.01 (0.63-4.28)	5.65 (1.90-10.86)	<.001	11.50 (8.00-28.75)	53.00 (38.00-86.87)	<.001	8.00 (7.31-18.45)	52.57 (20.89-86.34)	<.001	4.94 (3.11-6.98)	3.63 (1.96-5.80)	.43
Platelets, × 10^3^/µL	221.0 (175.0-284.5)	351.5 (271.5-457.8)	<.001	252.0 (198.8-320.2)	354.0 (300.5-461.5)	<.001	246.0 (21.01-304.3)	351.0 (284.0-417.0)	<.001	264.5 (227.8-318.0)	378.5 (304.8-456.0)	<.001	213.0 (171.0-274.8)	329.5 (286.2-431.5)	.03
WBC, cells/µL	7100 (5600-9800)	12 300 (10 500-14 000)	<.001	7300 (5600-9800)	12 800 (10 400-16 600)	<.001	9400 (6300-13 000)	12 200 (11 100-16 600)	<.001	8000 (6500-11 400)	12 800 (9400-16 900)	<.001	9050 (7600-10 450)	9700 (8430-12 980)	.25
Mature neutrophil, %	42 (33.5-58.0)	58.0 (43.3-70.5)	.007	46.8 (30.3-61.2)	58.8 (33.5-67.1)	<.001	72.0 (59.2-79.3)	64.2 (53.5-74.1)	.05	62.3 (52.5-72.2)	62.9 (49.7-74.8)	.56	57.5 (47.4-62.6)	65.2 (50.3-69.8)	.73
Immature neutrophil, %	0 (0-0)	0 (0-1)	.45	0 (0-0)	0 (0-0)	.62	NA	NA	NA	NA	NA	NA	0 (0-0)	0 (0-0)	.37
ANC, cells/µL	2900 (1800-4600)	7300 (4400-9300)	<.001	3100 (1700-6100)	7300 (5300-10 100)	<.001	NA	NA	NA	NA	NA	NA	4400 (3500-6400)	7400 (5000-8500)	.46
Heart lesion status															
Aneurysm	NA	10 (25.0)	<.001	NA	16 (26.7)	<.001	NA	3 (5.7)	<.001	NA	6 (7.5)	<.001	NA	2 (20.0)	<.001
Dilated coronary artery	NA	3 (7.5)		NA	4 (6.7)		NA	4 (7.5)		NA	2 (2.5)		NA	3 (30.0)	
Normal coronary artery	NA	27 (67.5)		NA	40 (66.7)		NA	46 (86.8)		NA	72 (90.0)		NA	5 (50.0)	
Incomplete KD	NA	7 (17.5)	<.001	NA	7 (11.7)	<.001	NA	14 (26.4)	<.001	NA	15 (18.8)	<.001	NA	6 (60.0)	<.001
FC diagnosis															
Bacterial	23 (57.5)	NA	NA	67 (55.4)	NA	NA	23 (28.4)	NA	NA	16 (34.8)	NA	NA	6 (60.0)	NA	<.001
Viral	2 (5.0)	NA	NA	6 (5.0)	NA	NA	28 (34.6)	NA	NA	18 (39.1)	NA	NA	1 (10.0)	NA	<.001
Mixed infection	1 (2.5)	NA	NA	9 (7.4)	NA	NA	0	NA	NA	1 (2.2)	NA	NA	0	NA	NA
Noninfection	14 (35.0)	NA	NA	39 (32.2)	NA	NA	30 (37.0)	NA	NA	11 (23.9)	NA	NA	30 (30.0)	NA	NA

Abbreviations: ANC, absolute neutrophil count; CRP, C-reactive protein; FC, febrile control; KD, Kawasaki disease; LN, lymph node; WBC, white blood cells.

SI conversion factors: To convert CRP to milligrams per liter, multiply by 10; hemoglobin to grams per liter, multiply by 10; neutrophils to proportion of 1.0, multiply by 0.01; platelets to ×10^9^/L, multiply by 1; WBC to cells/µL, multiple by 1000.

^a^ Pooled demographic, clinical, and laboratory characteristics across all cohorts are provided in eTable 14 in Supplement 1.

^b^ Taiwan cohorts, enrolling from June 2012 to November 2021.

^c^ Shanghai, China, cohorts, enrolling from November 2022 to June 2023.

^d^ Taiwan cohort, enrolling from November 2017 to March 2023. This cohort represents a targeted laboratory validation set and was not age-matched by design.

^e^ Clinical symptoms refer to principal KD features, including oral mucosal changes (lip/oral), bilateral nonexudative conjunctival injection (conjunctival), cervical lymphadenopathy (cervical LN), extremity changes (extremity), and polymorphous rash (rash).

Febrile control (FC) participants were age-comparable children hospitalized for alternative febrile illnesses, including confirmed viral infections (eg, influenza, respiratory syncytial virus), confirmed bacterial infections (eg, urinary tract infection), mixed infections, and noninfectious inflammatory conditions. FC diagnoses were established based on standard clinical, microbiologic, and laboratory evaluation at each site.

Exclusion criteria included prior treatment with intravenous immunoglobulin before sample collection, known chronic inflammatory or autoimmune disease, or inadequate RNA yield for molecular testing. During periods of routine SARS-CoV-2 surveillance, children meeting clinical criteria for multisystem inflammatory syndrome in children were adjudicated as alternative diagnoses and were not enrolled as KD cases.

### Reference Standard

The reference standard diagnosis of KD was based on the 2017 AHA criteria for complete or incomplete KD. All KD diagnoses were independently adjudicated by 2 board-certified pediatric cardiologists at each participating region (H.-C.K. and K.-P.W. in Taiwan; X.X. and F.L. in Shanghai).

Adjudication incorporated clinical features, laboratory findings, and echocardiographic data obtained at presentation and during follow-up, in accordance with AHA guidelines. Discrepancies were resolved by consensus review. Clinical adjudicators were blinded to index test results, and molecular testing was performed without access to reference standard classification, thereby minimizing incorporation and review bias.

### RT-qPCR Assay

Peripheral blood mononuclear cell–derived RNA from cohorts A to E was analyzed using a quantitative PCR assay measuring *IFI27* and *MCEMP1* expression (eFigures 2-8 in Supplement 1). Cycle threshold (Ct) values were normalized to glyceraldehyde 3-phosphate dehydrogenase, and a composite KD score was calculated using a prespecified equation. The assay was developed and analytically validated as a laboratory-developed test within a laboratory certified by the College of American Pathologists and Clinical Laboratory Improvement Amendments (CAP/CLIA) (eFigures 3-8 in Supplement 1).^20,21^ All testing and classification were performed under blinded conditions.

Laboratory personnel performing RNA extraction, reverse transcription qPCR, and score calculation were blinded to clinical diagnosis and reference standard adjudication. Additional details regarding assay development and analytical validation are provided in the eMethods in Supplement 1.

### Definition of KD Score and Diagnostic Thresholds

KD score was calculated as change in Ct for *IFI27* minus change in Ct *MCEMP1*, corresponding to the log_2_ expression ratio between the 2 genes. KD is characterized by relative suppression of *IFI27* and induction of *MCEMP1* compared with other febrile illnesses, resulting in higher KD scores (eFigure 9 and eFigure 10 in Supplement 1).

Diagnostic thresholds were defined in cohort A prior to evaluation in subsequent cohorts. A prespecified dual-threshold model was used to optimize clinical decision-making: 1 threshold targeted at least 95% negative predictive value (NPV) to support rule-out of KD, and a second targeted at least 95% positive predictive value (PPV) to support rule-in of KD within cohort A. Scores between these thresholds were classified as indeterminate, reflecting biological overlap rather than assay failure. This approach was designed to preserve high-confidence clinical decisions while avoiding forced binary classification in borderline cases and is consistent with our previously published KD diagnostic frameworks.^9,10,11,12^

These thresholds were locked before evaluation in independent validation cohorts (B-E) and were applied without modification. No reoptimization, recalibration, or refitting of the scoring equation or thresholds was performed in validation cohorts.

For primary binary performance analyses, samples with indeterminate scores were excluded from sensitivity and specificity calculations but were included in receiver operating characteristic (ROC) analyses and descriptive subgroup analyses to reflect intended clinical use.

### KD-Mimic Comparator Analysis

To evaluate diagnostic performance in clinically challenging presentations, a prespecified clinically enriched febrile control subgroup was constructed to simulate real-world diagnostic ambiguity. Febrile controls were classified as KD mimics (eFigure 6 in Supplement 1) if they met at least 1 of the following criteria at presentation: prolonged fever with at least 1 principal KD clinical feature, a high-inflammatory phenotype (defined by C-reactive protein [CRP] ≥3 mg/dL [to convert to milligrams per liter, multiple by 10] with neutrophil predominance, defined as absolute neutrophil count 7500 cells/µL [to convert to × 10^9^/L, multiply by 0.001] or ≥70% [to convert to proportion of 1.0, multiply by 0.01]), or laboratory-confirmed adenovirus infection.

Diagnostic performance within this KD-mimic subgroup was evaluated using the same locked scoring equation and diagnostic thresholds defined in cohort A. No recalibration or threshold modification was performed for this analysis.

### Analytical Validation

The assay was implemented as a CAP-accredited, CLIA-certified laboratory-developed test. Analytical performance met predefined criteria (eFigures 2-8 and eTables 6-13 in Supplement 1), with details provided in the eMethods in Supplement 1.

### Statistical Analysis

Continuous variables are reported as mean (SD) or median (IQR), as appropriate. Categorical variables are presented as counts and percentages. Group comparisons were performed using the *t* test or Wilcoxon rank-sum test for continuous variables and the χ^2^ test or Fisher exact test for categorical variables, as appropriate.

Diagnostic performance of the KD score was assessed using ROC curve analysis. Area under the ROC (AUC) values and corresponding 95% CIs were calculated using the DeLong method. Sensitivity, specificity, PPV, NPV, and positive and negative likelihood ratios were calculated with 95% CIs based on exact binomial methods.

Primary binary performance analyses were conducted using the prespecified locked thresholds. No recalibration, refitting, or modification of the scoring equation or thresholds was performed in validation cohorts. Samples classified as indeterminate were excluded from sensitivity and specificity calculations but were included in ROC analyses and descriptive subgroup analyses. Multivariable logistic regression was performed to evaluate whether the KD score remained independently associated with KD after adjustment for age, sex, and CRP. Odds ratios (ORs) with 95% CIs were reported. Missing data accounted for less than 5% of observations and were handled using complete-case analysis without imputation.

Because predictive values depend on disease prevalence, PPVs and NPVs were modeled across a range of assumed KD prevalence scenarios using standard bayesian formulas based on the observed sensitivity and specificity from the primary locked-threshold analysis. Prevalence settings were selected to reflect clinically relevant diagnostic contexts in which KD is evaluated, including febrile emergency department populations, hospitalized pediatric febrile evaluations, and tertiary referral settings in which KD is actively suspected, based on previously reported epidemiologic and clinical cohort studies.^3,9,10,11,22,23^

*P* values were 2-sided, and statistical significance was set at *P* < .05. All statistical analyses were performed using R software version 4.3 (R Foundation for Statistical Computing). Data were analyzed from January 2022 to August 2025.

## Results

### Study Population

Among 600 children screened for eligibility, 541 (90%; mean [SD] age, 3.7 [1.9] years; 300 [55.5%] male) met inclusion criteria and had adequate RNA available for testing (Figure 1). Fifty-nine patients were excluded due to incomplete clinical data or insufficient RNA quality.

The final analytic cohort included 243 patients with KD, of whom 194 (80%) had complete KD and 49 (20%) had incomplete KD, and 298 febrile controls. Baseline demographic, clinical, and laboratory characteristics by cohort are summarized in Table 1, and pooled characteristics for the overall analytic cohort are provided in eTable 14 in Supplement 1. Detailed etiologic classifications of febrile control diagnoses are provided in eTable 5 and eTable 15 in Supplement 1.

### Primary Diagnostic Performance

**Figure 2.**
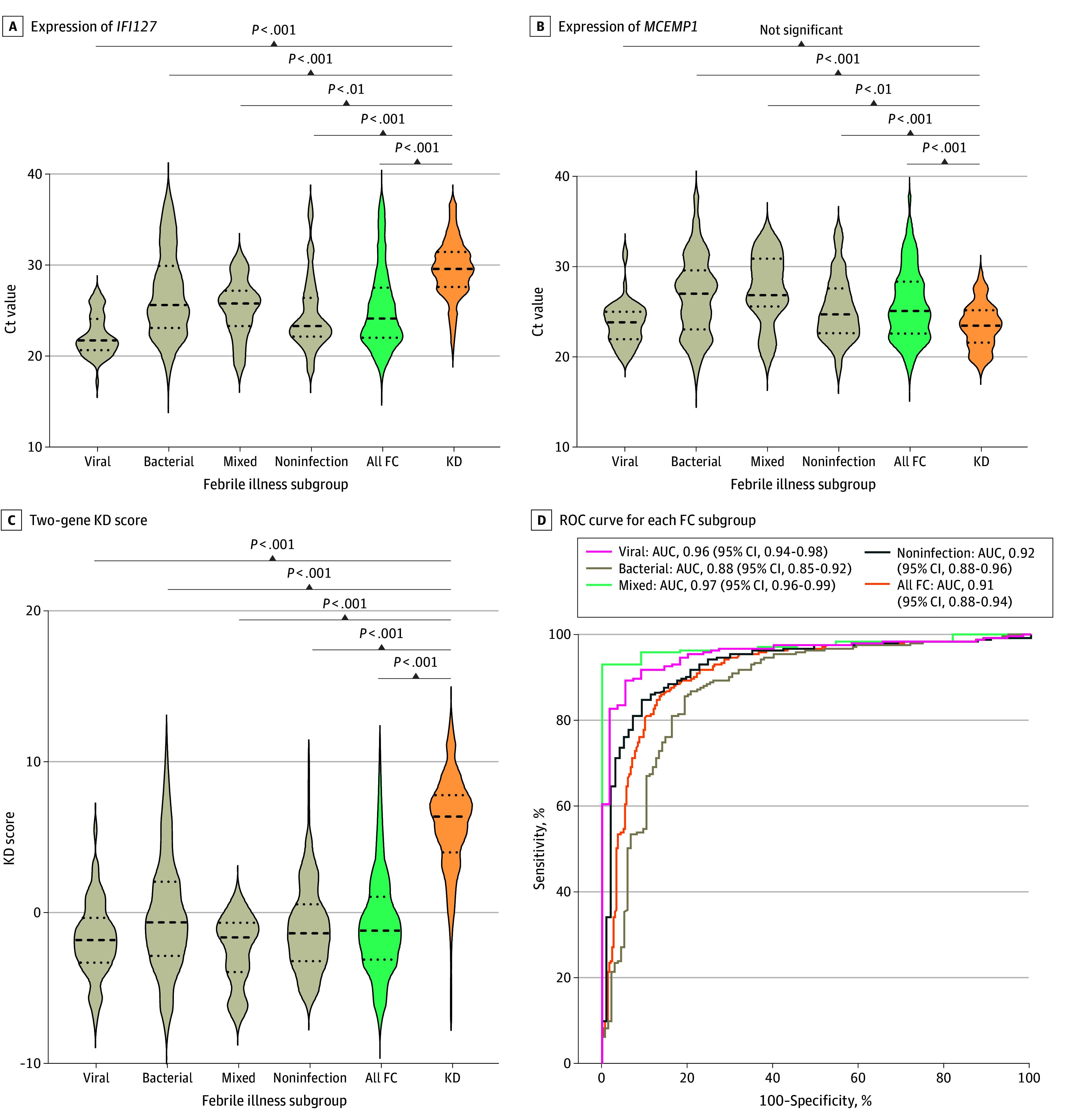
Violin Plot of Discrimination of Common Pediatric Bacterial, Virus, Mixed Infection, and Noninfection Febrile Illness From Kawasaki Disease (KD) Expression of *IFI27* (A) and *MCEMP1* (B), and the 2-gene KD score (C) effectively differentiates KD from other pediatric conditions. Receiver operating characteristic (ROC) curves were generated for each febrile control (FC) subgroup compared with KD with area under the ROC curve and 95% CI (D). *P* values were calculated with *t* test.

Across the full analytic cohort, the *IFI27-MCEMP1* KD score distinguished KD from febrile illnesses with an AUC of 0.91 (95% CI, 0.88-0.94) (Figure 2). Using prespecified locked thresholds, sensitivity was 94% (95% CI, 91%-97%) and specificity was 82% (95% CI, 78%-86%). The PPV was 84% (95% CI, 70%-94%), and the NPV was 94% (95% CI, 83%-99%), corresponding to the observed KD prevalence of 45% in the analytic cohort. Because predictive values depend on disease prevalence, modeled PPVs and NPVs across clinically relevant KD prevalence settings are shown in Table 2, with detailed point estimates across a broader range of assumed prevalences provided in eTable 16 in Supplement 1. The positive likelihood ratio was 5.12 (95% CI, 4.78-5.29), and the negative likelihood ratio was 0.05 (95% CI, 0.03-0.1).

**Table 2.** Modeled Predictive Values Across Clinically Relevant KD

Clinical context	Range, %		
	Assumed KD prevalence^a^	Modeled PPV^b^	Modeled NPV^b^
Population incidence context^c^	0.02-0.08	0.10-0.42	99.99-99.99
Febrile emergency department population	1-3	5.0-13.9	99.7-99.9
Hospitalized pediatric febrile evaluation	5-10	21.6-36.7	99.2-99.6
Children referred for suspected KD evaluation	20-50	56.6-83.9	93.2-98.2

Abbreviations: KD, Kawasaki disease; NPV, negative predictive value; PPV, positive predictive value.

^a^ Prevalence settings represent clinically relevant settings in which KD is evaluated, including febrile emergency department populations, hospitalized pediatric febrile evaluations, and tertiary referral populations where KD is actively suspected. Detailed point estimates for each assumed prevalence (0.02%, 0.08%, 1%, 3%, 5%, 10%, 20%, and 50%) are provided in eTable 16 in Supplement 1.

^b^ PPV and NPV were modeled using the pooled sensitivity (0.94) and specificity (0.82) derived from the locked-threshold analysis. PPV and NPV ranges summarize modeled values across clinically relevant KD prevalence settings.

^c^ Population incidence values are shown for context only and do not represent the intended use of the test. Prevalence ranges were informed by published epidemiologic and clinical cohort studies.

Using the prespecified dual-threshold model, 96 of 541 participants (17.7%) were classified as indeterminate. Among 243 patients with KD, 26 (10.7%) fell within the indeterminate range, compared with 70 of 298 febrile controls (23.5%). Among 445 determinate results, sensitivity and specificity were 0.96 (95% CI, 0.93-0.98) and 0.82 (95% CI, 0.76-0.86), respectively. No patients with coronary artery dilation were misclassified as febrile controls.

### Validation Across Independent Cohorts

Diagnostic performance was consistent across the 5 independent cohorts (A-E), with cohort-level AUCs ranging from 0.90 to 0.95 and no evidence of between-cohort heterogeneity (*I*^2^ = 0.0% [95% CI, 0.0%-67.5%]; Cochran *Q* = 2.66; *df* = 4; *P* = .62) (Figure 2; eFigure 2, eFigure 11, and eFigure 12 in Supplement 1). Diagnostic thresholds defined in cohort A were applied without modification in all subsequent cohorts.

In blinded validation cohorts, AUCs were 0.90 (95% CI, 0.86-0.95) in cohort B, 0.92 (95% CI, 0.88-0.97) in cohort C, and 0.90 (95% CI, 0.85-0.96) in cohort D. In cohort E, where the assay was implemented as a CAP-accredited, CLIA-certified laboratory-developed test, the AUC was 0.91 (95% CI, 0.82-0.93).

### Expression Patterns and Biological Directionality

Violin plots demonstrated reciprocal gene expression patterns in patients with KD compared with febrile controls (Figure 2). *IFI27* expression was suppressed, whereas *MCEMP1* expression was elevated in KD, resulting in higher composite KD scores. This reciprocal pattern produced clear separation between KD and viral, bacterial, mixed, and noninfectious febrile subgroups (Figure 2).

### Subgroup Analyses

**Figure 3.**
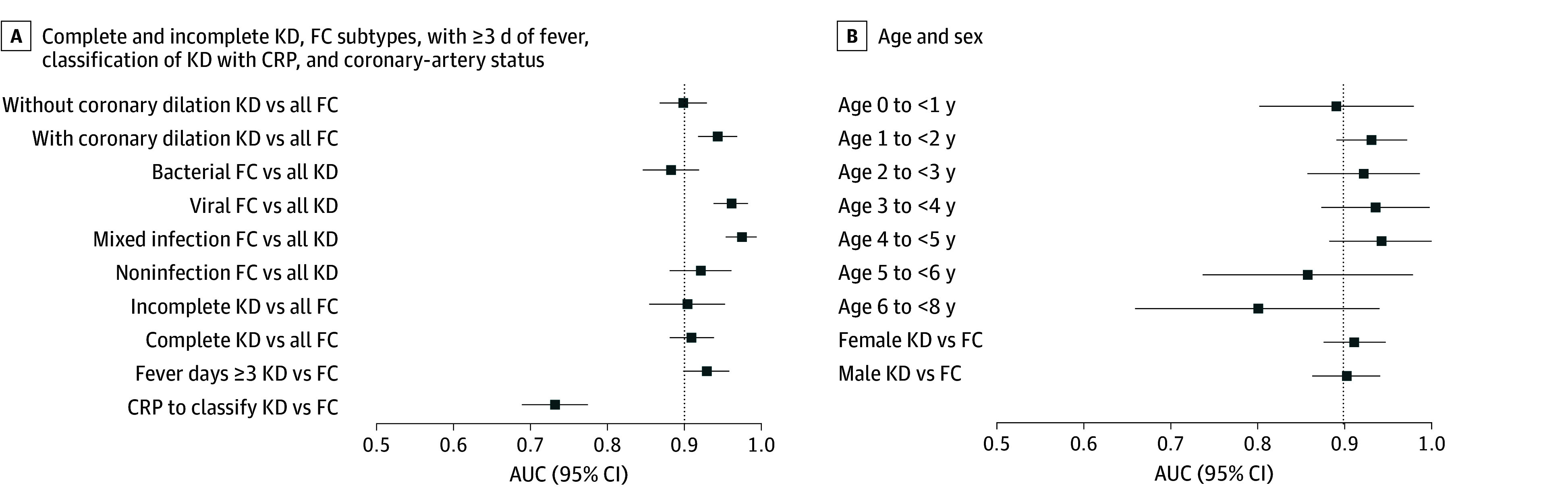
Dot and Whisker Plots of the Diagnostic Performance of the Kawasaki Disease (KD) Score Across Clinical Subgroups and Comparison With C-Reactive Protein (CRP) The vertical dashed lines indicate reference area under the receiver operating characteristic curve (AUC) of 0.90. FC indicates febrile control.

Diagnostic performance remained stable across clinically relevant subgroups (Figure 3; eFigures 13-19 in Supplement 1). Discrimination was similar for complete and incomplete KD, with AUCs ranging from 0.89 to 0.91. Performance was also consistent across febrile control subtypes, including viral, bacterial, mixed, and noninfectious conditions.

The KD score demonstrated comparable discrimination across age strata and sex, with AUCs ranging from 0.86 to 0.95. These findings indicate stable performance across diverse clinical presentations.

### Independence From Inflammatory Markers and Clinical Covariates

Although CRP levels were elevated across febrile illnesses, they were significantly higher in patients with KD than in febrile controls (eFigure 14 in Supplement 1). In multivariable logistic regression adjusting for age, sex, and CRP, the KD score remained independently associated with KD (OR, 7.6 [95% CI, 5.1-11.3]; *P* < .001). The KD score demonstrated superior discrimination compared with CRP alone (AUC, 0.91 [95% CI, 0.88-0.94] vs 0.73 [95% CI, 0.69-0.78]; *P* < .001), indicating that its diagnostic performance was not attributable solely to nonspecific systemic inflammation.

### Coronary Artery Findings

Among 37 patients with coronary artery aneurysms, 35 (94.6%) were correctly classified as having KD using prespecified thresholds; the remaining 2 patients were assigned to the indeterminate range, and none were misclassified as febrile controls. All patients with coronary artery dilation were classified as having KD or indeterminate, with no false-negative results (eFigure 19 in Supplement 1). These findings indicate preserved diagnostic sensitivity among patients with clinically significant coronary involvement.

### Performance in Clinically Enriched KD-Mimic Presentations

In a prespecified clinically enriched KD-mimic subgroup designed to simulate real-world diagnostic ambiguity, the KD score retained robust performance using locked thresholds (eFigure 18 in Supplement 1). Among 217 patients with determinate results, 208 patients with KD (95.9%) were correctly classified, and misclassification of KD as febrile control was uncommon (9 patients [4.1%]). Diagnostic uncertainty was largely confined to the predefined indeterminate range, supporting its role as a safety buffer rather than a diagnostic failure.

### Modeling Predictive Values Across Clinically Relevant KD Prevalence Settings

Because predictive values depend on disease prevalence, the PPVs and NPVs observed in this tertiary referral population may not generalize to lower-prevalence settings. Therefore, we modeled predictive values across clinically relevant KD prevalence settings using the observed sensitivity and specificity (Table 2; eTable 16 in Supplement 1).

## Discussion

In this multicenter diagnostic study of febrile children, a 2-gene blood test measuring *IFI27* and *MCEMP1* expression distinguished KD from other febrile illnesses with high accuracy (AUC, 0.91) and sensitivity using prespecified locked thresholds. Diagnostic performance was consistent across independent cohorts, including complete and incomplete KD, diverse infectious etiologies, and varying age and sex strata. Importantly, no patients with coronary artery dilation were misclassified as febrile controls, and most patients with coronary aneurysms were correctly identified or assigned to the indeterminate range. These findings support the *IFI27*-*MCEMP1* score as a reproducible molecular adjunct to clinical evaluation in children with suspected KD.

Diagnostic uncertainty remains a major challenge in KD, particularly in children presenting with incomplete or overlapping features of viral or bacterial infection. Because delayed recognition is associated with increased risk of coronary artery complications, diagnostic tools with high sensitivity and NPV are clinically valuable. The consistently high sensitivity observed in this study suggests that the *IFI27*-*MCEMP1* assay may function as an adjunctive rule-out test in diagnostically uncertain presentations. In practice, integration of a quantitative molecular score could support earlier echocardiographic evaluation and more timely treatment decisions when clinical criteria alone are insufficient.

In line with our prior work,^9,10,11,12^ the dual-threshold framework was intentionally designed to prioritize high-confidence clinical decisions rather than force binary classification in biologically overlapping cases. Using prespecified thresholds targeting at least 95% PPV and NPV, 17.7% of patients were classified as indeterminate. Importantly, this intermediate category was not disproportionately concentrated among patients with KD and did not materially reduce diagnostic sensitivity. Instead, diagnostic uncertainty was preferentially captured within the indeterminate zone rather than resulting in unsafe false-negative classifications, as no patients with coronary artery dilation were misclassified as febrile controls. In practice, such intermediate results would prompt continued monitoring or repeat evaluation, consistent with current guideline-based management of incomplete or evolving KD.

Prior transcriptomic investigations have reported multigene expression signatures for KD, including a 13-gene classifier and other host-response panels.^16^ These studies demonstrated the biological feasibility of transcriptomic discrimination but were primarily discovery-focused and not uniformly validated under blinded, multicenter clinical conditions. In contrast, this study evaluated a parsimonious 2-gene assay using prespecified scoring criteria applied across independent cohorts. Despite its simplicity, the assay achieved diagnostic performance comparable to previously reported multigene panels, supporting the feasibility of reduced gene signatures for clinical deployment.

The reciprocal expression patterns of *IFI27* and *MCEMP1* provide biological plausibility for the diagnostic score. *IFI27*, an interferon-stimulated gene,^24^ was relatively suppressed in KD compared with infectious febrile illnesses, whereas *MCEMP1*, associated with myeloid and mast cell activation, was elevated.^25,26^ This pattern suggests an immune profile distinct from typical antiviral or bacterial inflammatory responses. The absence of false-negative classification among patients with coronary artery dilation and the high detection rate among those with aneurysms are consistent with the possibility that this immune signature reflects clinically significant disease activity.

Prospective studies in emergency and outpatient settings are needed to determine whether integration of the *IFI27*-*MCEMP1* score into diagnostic workflows improves time to treatment, echocardiography utilization, or coronary outcomes. Across lower-prevalence environments, such as febrile emergency department populations, the NPVs remained very high (>98%), supporting potential clinical utility as an adjunctive rule-out tool to reduce diagnostic uncertainty. In contrast, in higher pretest probability settings where KD was actively suspected, the PPVs increased substantially, supporting potential rule-in utility in referral populations.

Evaluation in more diverse populations and across broader inflammatory conditions will further clarify generalizability. As rapid molecular platforms become increasingly available, simplified host-response assays may complement clinical criteria in the evaluation of suspected KD.

### Strengths and Limitations

This study has several strengths. Participants were prospectively enrolled across multiple tertiary centers, with blinded adjudication of the reference standard and blinded molecular testing. Diagnostic thresholds were defined prior to validation and applied without recalibration across cohorts. Diagnostic performance remained stable across geographically and clinically distinct cohorts, with minimal between-cohort heterogeneity, supporting the robustness of the locked diagnostic thresholds across differing patient populations. The assay was implemented within a CAP-accredited, CLIA-certified laboratory, supporting analytical robustness and potential clinical scalability.

This study has limitations. First, although samples were prospectively collected, validation cohorts were assembled retrospectively, and the KD prevalence in the analytic cohort may not reflect prevalence in other settings, potentially influencing predictive values. Second, clinical validation was limited to East Asian populations; performance in other geographic or genetic backgrounds requires further evaluation. Third, the number of patients with incomplete KD was modest, and additional validation in larger incomplete cohorts is warranted. Fourth, specificity varied modestly across sites, highlighting the need for standardized calibration procedures across laboratories. Fifth, not all inflammatory or autoimmune disorders were represented among febrile controls, which may affect specificity. Sixth, as with any RNA-based assay, preanalytical variables may influence reproducibility, although predefined stability and performance criteria were met.

## Conclusions

This diagnostic study found that a 2-gene whole-blood reverse transcription qPCR assay demonstrated consistent diagnostic accuracy for distinguishing KD from other febrile illnesses across multiple centers. When applied using prespecified locked thresholds, the assay showed high sensitivity and stable performance across subgroups. These findings support its potential role as an adjunct to clinical evaluation in children with suspected KD, pending further prospective implementation studies.

## Notes

Supplement 1. eMethods. eFigure 1. Discovery and screening of optimized 2-gene pairs for differential expression in Kawasaki 30 Disease eFigure 2. Clinical and analytical validation for a laboratory developed test eFigure 3. Accuracy of the *IFI27* and *MCEMP1* assays eFigure 4. Linearity of *IFI27* and *MCEMP1* assays eFigure 5. *IFI27* and *MCEMP1* have good precision performance eFigure 6. Assays detect specific RNA for *IFI27* and *MCEMP1* eFigure 7. gDNA of *MCEMP1* has a distinct shifted melting temperature eFigure 8. Portability of RT-qPCR eFigure 9. Individual gene validation transition from Microarray to RNAseq to RT-qPCR eFigure 10. KD score validation in Microarray, RNAseq and RT-qPCR eFigure 11. KD assessment with cohort A and diagnostic threshold determination eFigure 12. KD assessment with cohort B, C, and D eFigure 13. KD score distinguishes pediatric febrile illness from KD eFigure 14. Distribution of CRP values across febrile control subgroups and Kawasaki disease and diagnostic performance of CRP as a comparator biomarker eFigure 15. The KD score separates KD patients from FC with reported KD signs eFigure 16. The KD score identifies complete and incomplete KD eFigure 17. The KD score performance similar cross age and sex eFigure 18. Construction of the clinically defined KD-mimic cohort and identification of KD from clinically defined KD-mimics and performance evaluation eFigure 19. KD test performance in aneurysm vs non-aneurysm KD eTable 1. Kawasaki disease and vasculitis datasets included in the HMS data mining algorithm eTable 2. 13 Pathways with associated genes eTable 3. 54 Gene candidates eTable 4. Top 20 gene pairs eTable 5. Clinical diagnoses of FC patients eTable 6. Clinical Characteristics for patients in the LDT validation eTable 7. Sensitivity/LLOQ of *IFI27* and *MCEMP1* assays eTable 8. Genomic DNA RT-qPCR results for *IFI27* and *MCEMP1* eTable 9. Genomic DNA results with and without rDNase treatment eTable 10. Whole blood RNA results with and without rDNase treatment eTable 11. Interfering substances testing concentrations eTable 12. Interfering substances do not affect assay results eTable 13. Whole blood sample storage RT-qPCR results eTable 14. Demographic/clinical characteristics and lab test results for patients with Kawasaki disease and febrile controls in this study eTable 15. Kawasaki disease indications in febrile controls eTable 16. Modeled positive and negative predictive values of the *IFI27*–*MCEMP1* assay across a 69 range of assumed Kawasaki disease prevalences eReferences.

Supplement 2. Data Sharing Statement
