# Supplementary material for: Validation of a 2-Gene Blood Test for Kawasaki Disease in Febrile Children
Source: JAMA Netw Open. 2026 May 28;9(5):e2615420. doi: 10.1001/jamanetworkopen.2026.15420 (PMC13220111; doi:10.1001/jamanetworkopen.2026.15420)
Supplement: Supplement 1. — eMethods. eFigure 1. Discovery and screening of optimized 2-gene pairs for differential expression in Kawasaki 30 Disease eFigure 2. Clinical and analytical validation for a laboratory developed test eFigure 3. Accuracy of the IFI27 and MCEMP1 assays eFigure 4. Linearity of IFI27 and MCEMP1 assays eFigure 5. IFI27 and MCEMP1 have good precision performance eFigure 6. Assays detect specific RNA for IFI27 and MCEMP1 eFigure 7. gDNA of MCEMP1 has a distinct shifted melting temperature eFigure 8. Portability of RT-qPCR eFigure 9. Individual gene validation transition from Microarray to RNAseq to RT-qPCR eFigure 10. KD score validation in Microarray, RNAseq and RT-qPCR eFigure 11. KD assessment with cohort A and diagnostic threshold determination eFigure 12. KD assessment with cohort B, C, and D eFigure 13. KD score distinguishes pediatric febrile illness from KD eFigure 14. Distribution of CRP values across febrile control subgroups and Kawasaki disease and diagnostic performance of CRP as a comparator biomarker eFigure 15. The KD score separates KD patients from FC with reported KD signs eFigure 16. The KD score identifies complete and incomplete KD eFigure 17. The KD score performance similar cross age and sex eFigure 18. Construction of the clinically defined KD-mimic cohort and identification of KD from clinically defined KD-mimics and performance evaluation eFigure 19. KD test performance in aneurysm vs non-aneurysm KD eTable 1. Kawasaki disease and vasculitis datasets included in the HMS data mining algorithm eTable 2. 13 Pathways with associated genes eTable 3. 54 Gene candidates eTable 4. Top 20 gene pairs eTable 5. Clinical diagnoses of FC patients eTable 6. Clinical Characteristics for patients in the LDT validation eTable 7. Sensitivity/LLOQ of IFI27 and MCEMP1 assays eTable 8. Genomic DNA RT-qPCR results for IFI27 and MCEMP1 eTable 9. Genomic DNA results with and without rDNase treatment eTable 10. Whole blood RNA results with and without rDNase treatment eT [file jamanetwopen-e2615420-s001.pdf]

Supplemental Online Content

Kuo H, Xue X, Liu F, et al. Validation of a 2-gene blood test for Kawasaki disease in febrile children. *JAMA Netw Open*. 2026;9(5):e2615420. doi:10.1001/jamanetworkopen.2026.15420

eMethods.
eFigure 1. Discovery and screening of optimized 2-gene pairs for differential expression in Kawasaki 30 Disease
eFigure 2. Clinical and analytical validation for a laboratory developed test
eFigure 3. Accuracy of the IFI27 and MCEMP1 assays
eFigure 4. Linearity of IFI27 and MCEMP1 assays
eFigure 5. IFI27 and MCEMP1 have good precision performance
eFigure 6. Assays detect specific RNA for IFI27 and MCEMP1
eFigure 7. gDNA of MCEMP1 has a distinct shifted melting temperature
eFigure 8. Portability of RT-qPCR
eFigure 9. Individual gene validation transition from Microarray to RNAseq to RT-qPCR
eFigure 10. KD score validation in Microarray, RNAseq and RT-qPCR
eFigure 11. KD assessment with cohort A and diagnostic threshold determination
eFigure 12. KD assessment with cohort B, C, and D
eFigure 13. KD score distinguishes pediatric febrile illness from KD
eFigure 14. Distribution of CRP values across febrile control subgroups and Kawasaki disease and diagnostic performance of CRP as a comparator biomarker
eFigure 15. The KD score separates KD patients from FC with reported KD signs
eFigure 16. The KD score identifies complete and incomplete KD
eFigure 17. The KD score performance similar cross age and sex
eFigure 18. Construction of the clinically defined KD-mimic cohort and identification of KD from clinically defined KD-mimics and performance evaluation
eFigure 19. KD test performance in aneurysm vs non-aneurysm KD
eTable 1. Kawasaki disease and vasculitis datasets included in the HMS data mining algorithm
eTable 2. 13 Pathways with associated genes
eTable 3. 54 Gene candidates
eTable 4. Top 20 gene pairs
eTable 5. Clinical diagnoses of FC patients
eTable 6. Clinical Characteristics for patients in the LDT validation
eTable 7. Sensitivity/LLOQ of IFI27 and MCEMP1 assays
eTable 8. Genomic DNA RT-qPCR results for IFI27 and MCEMP1
eTable 9. Genomic DNA results with and without rDNase treatment
eTable 10. Whole blood RNA results with and without rDNase treatment
eTable 11. Interfering substances testing concentrations
eTable 12. Interfering substances do not affect assay results
eTable 13. Whole blood sample storage RT-qPCR results
eTable 14. Demographic/clinical characteristics and lab test results for patients with Kawasaki disease and febrile controls in this study
eTable 15. Kawasaki disease indications in febrile controls
eTable 16. Modeled positive and negative predictive values of the IFI27–MCEMP1 assay across a 69 range of assumed Kawasaki disease prevalences
eReferences.

This supplemental material has been provided by the authors to give readers additional information about their work.

© 2026 Kuo H et al. *JAMA Network Open*.

© 2026 Kuo H et al. *JAMA Network Open*.

eMethods.

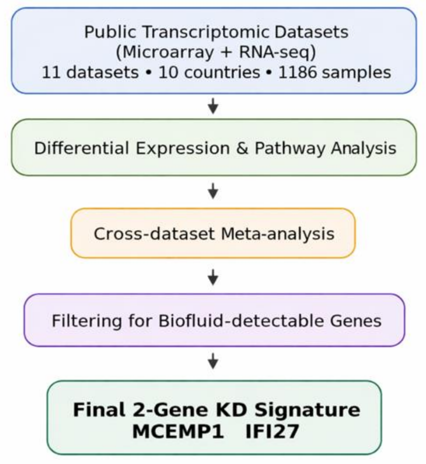

Discovery and Validation Overview

We conducted a meta-analysis of 11 publicly available whole-blood transcriptomic datasets (microarray and RNA sequencing) comprising 527 patients with Kawasaki disease (KD), 623 febrile controls (FCs), and 36 patients with other vasculitides from 10 countries (eFigure 1; eTable 1). For each dataset, gene-level log fold changes comparing KD and FC samples were computed and combined using a random-effects model to account for between-study heterogeneity.

All preprocessing and meta-analytic procedures were performed exclusively for biomarker discovery and were not applied to clinical validation cohorts. Datasets containing healthy controls or other inflammatory conditions were included only for biological contrast during candidate prioritization and were not used in diagnostic performance estimation.

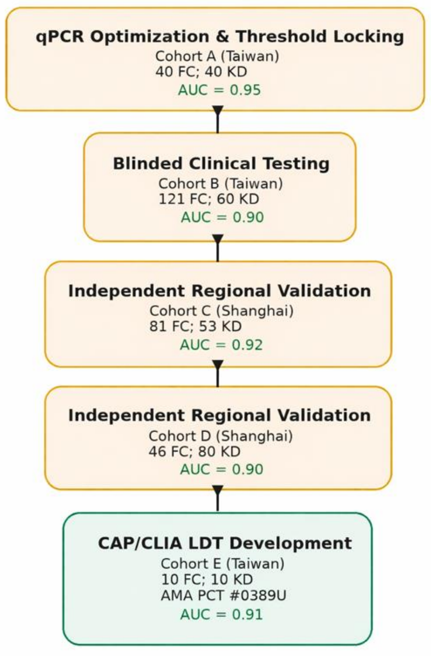

Genes were ranked using a composite metric integrating pooled effect size and interstudy heterogeneity, favoring markers with consistent differential expression across platforms and populations. From this analysis, IFI27 and MCEMP1 emerged as the most reproducible diagnostic gene pair across datasets, demonstrating strong discriminatory signal with minimal platform dependence (eFigures 2–3; eTable 2–4).

Following discovery, a prospective multicohort clinical validation study was conducted. Cohort A (Taiwan) was used for assay calibration and diagnostic threshold definition. These thresholds were locked prior to testing in independent cohorts. Blinded validation was performed in Cohort B (Taiwan) and independently replicated in Cohorts C and D (Shanghai). Cohort E (Taiwan) reflects implementation as a CAP-accredited, CLIA-certified laboratory-developed test (AMA PLA code PCT #0389U). Diagnostic performance across validation cohorts was summarized using area under the receiver operating characteristic curve (AUC).

Multicohort transcriptomic discovery of a two-gene diagnostic panel

To identify a robust molecular signature for Kawasaki disease (KD), we applied the Hyperflex-Meta-Signature (HMS) platform, a customized multi-cohort bioinformatics pipeline, to a curated collection of 11 publicly available transcriptomic datasets. These datasets encompassed 1,186 blood samples from pediatric patients across 10 countries (eTable 1) and were stratified into three analytic tiers: a discovery cohort (Cohort 1, n = 204) for gene selection, a validation cohort (Cohort 2, n = 595) for internal performance assessment, and a technical replication cohort (Cohort 3, n = 387) used for cross-platform confirmation. The HMS pipeline integrated statistical significance, effect size, and biological relevance through a composite scoring system to prioritize differentially expressed genes. This approach identified IFI27 and MCEMP1 as the top-performing gene pair for distinguishing KD from febrile controls.

Experimental Design

This study was designed to test the hypothesis that Kawasaki disease (KD) can be distinguished from other pediatric febrile illnesses by a reproducible gene expression signature that reflects underlying KD-specific immune dysregulation and vascular inflammation. To identify and validate such a signature, we employed a multi-stage approach combining *in silico* meta-analysis, RT-qPCR assay development, and clinical laboratory validation.

© 2026 Kuo H et al. *JAMA Network Open*.

The initial discovery phase utilized the Hyperflex-Meta-Signature (HMS) platform—a modular, multi-cohort bioinformatics pipeline developed for systematic meta-analysis of publicly available gene expression microarray and RNA-seq datasets. The HMS algorithm integrates pathway-level enrichment, cross-cohort harmonization, and candidate gene prioritization based on fold-change, statistical significance, and detectability in peripheral blood. By comparing transcriptomic profiles across multiple patient groups, including KD, other vasculitides, and febrile controls, the pipeline identified genes with discriminatory potential. The gene selection and prioritization process is described in detail below.

Candidate genes from the HMS analysis were then evaluated in a series of nested case-control RT-qPCR validation cohorts comprising PBMC-derived RNA samples from clinically confirmed KD cases and febrile illness controls. These validation studies were conducted under blinded conditions and spanned multiple geographic sites. Analytical validation of the assay was performed in a College of American Pathologists (CAP)-accredited clinical laboratory in the United States.

Sample size for RT-qPCR validation was determined by power analysis using the method described by Akoglu et al [1]. Assuming a target sensitivity of 85% and specificity of 95%, with initial estimates from Cohort A, a minimum sample size of 80 was required to achieve a marginal error below 10%. Inclusion and exclusion criteria, sample handling, blinding procedures, and validation methodologies are detailed in subsequent sections.

© 2026 Kuo H et al. *JAMA Network Open*.

Methods

Hyperflex-Meta-Signature (HMS) platform for biomarker discovery

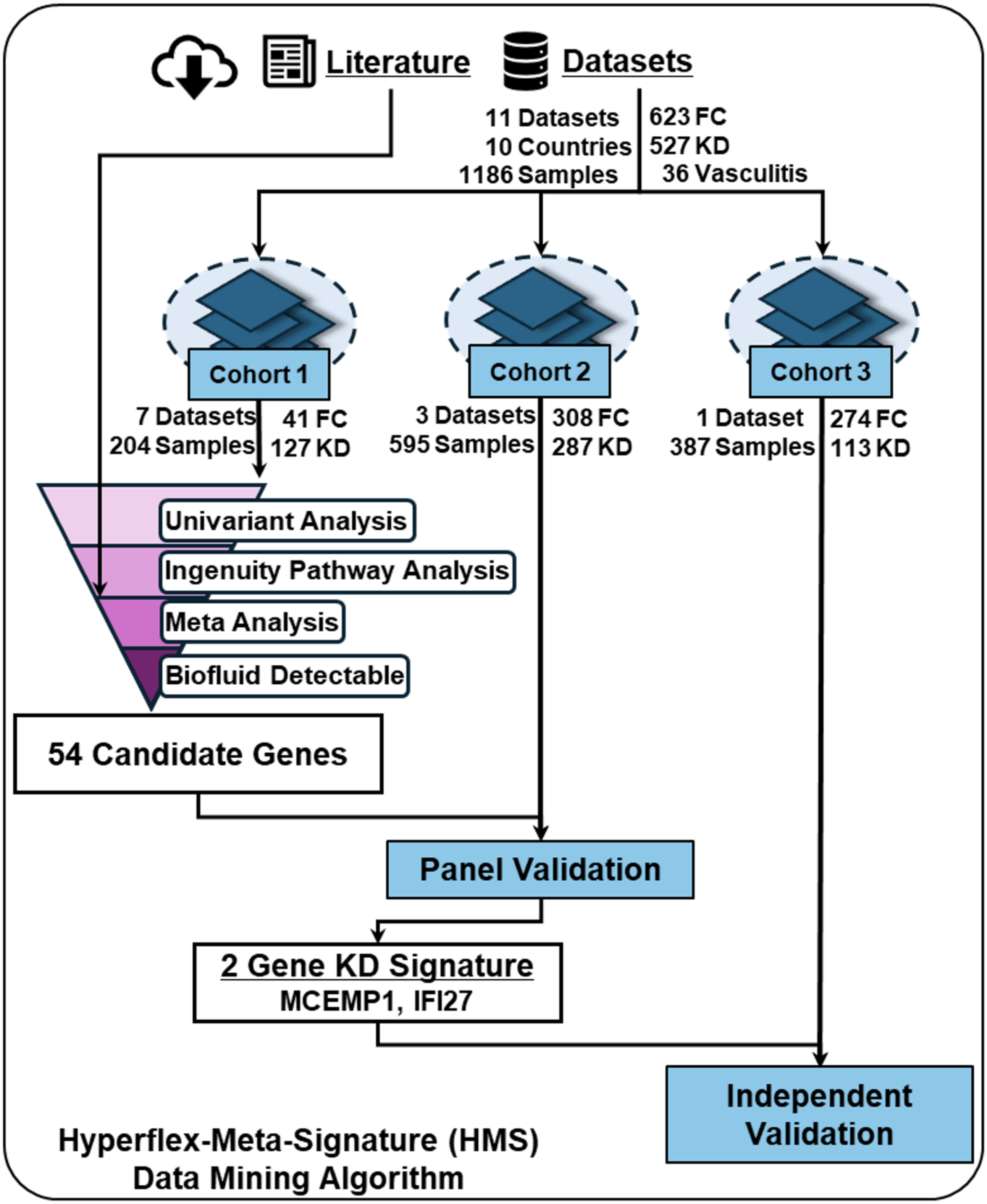

We developed the Hyperflex-Meta-Signature (HMS) platform as a modular pipeline to integrate heterogeneous transcriptomic datasets and identify robust candidate biomarkers for Kawasaki disease (KD). The HMS workflow includes cross-platform normalization, pathway activity profiling, and multi-metric feature prioritization.

© 2026 Kuo H et al. *JAMA Network Open*.

Data preprocessing and normalization:

Gene expression datasets from microarray platforms (GPL8328, GPL570, GPL20301) were $\log_2$-transformed (if needed) and quantile-normalized within platform. RNA-seq data were normalized using the trimmed mean of M-values (TMM) method via the edgeR package, then $\log_2$-transformed to counts per million (CPM). To integrate datasets across platforms, batch effects were mitigated using the ComBat empirical Bayes method[2], preserving biological variation while reducing technical noise.

Pathway activity and signature consistency analysis:

We assessed biological concordance across cohorts by calculating pathway activity scores using curated gene sets. Variability in pathway activation was evaluated across datasets to identify consistently perturbed biological processes. Genes contributing to high-consistency pathways were retained for candidate prioritization.

Feature prioritization and composite scoring:

Candidate gene markers were ranked using a composite significance score integrating statistical significance (from t-tests or Mann-Whitney U tests) and biological relevance (fold-change magnitude), as described by Xiao et al [3]. This approach enabled identification of robust individual genes and gene-pair ratios showing strong discriminatory power across KD and febrile control samples.

Computational performance and scalability:

The HMS pipeline was implemented on a high-performance server (dual Intel Xeon CPUs, 1 TB RAM). For datasets comprising ~1,000 samples, the full workflow—including normalization, batch correction, feature ranking, and output generation—completed in approximately five minutes. The modular architecture enables efficient scaling for larger datasets without substantial increases in runtime.

Gene expression datasets for KD and vasculitis discovery cohorts

To identify candidate biomarkers for Kawasaki disease (KD), we applied the HMS pipeline to publicly available whole blood or PBMC gene expression datasets sourced from the NCBI Gene Expression Omnibus (GEO). These datasets included patient samples from three disease categories: KD, febrile controls (FC), and other immune-mediated vasculitides. The objective was to identify differentially expressed genes and pathway-level features capable of distinguishing KD from both non-vasculitic febrile illnesses and other forms of vasculitis.

We assembled 11 transcriptomic datasets into three composite cohorts: Cohort 1 (discovery), consisting of 204 samples; Cohort 2 (cross-validation), consisting of 595 samples; and Cohort 3 (independent validation), consisting of 387 samples, as detailed in eTable 1. Among these datasets, four directly profiled KD patients. One dataset (GSE15297) compared KD with febrile controls, while three others (GSE18606, GSE9864, and GSE9863) compared KD to healthy individuals. To extend our analysis to other forms of vasculitis, we included datasets profiling Takayasu's arteritis (GSE33910 and GSE16945) and Behçet's disease (GSE17114).

KD is a systemic, acute vasculitis that predominantly affects medium-sized arteries in infants and young children. Because TA and BD are also primary immune-mediated vasculitides characterized by vascular inflammation, endothelial dysfunction, and immune cell infiltration, we reasoned that their inclusion would help distinguish KD-specific molecular signatures from more general features of vasculitic inflammation. This comparative strategy allowed us to isolate transcriptional signals uniquely enriched in KD.

To prepare the datasets for integration, Ensembl gene identifiers were first converted to Entrez gene IDs to ensure consistency across platforms. Matched gene and sample identifiers were then used to merge data from different studies before normalization and batch effect correction. These harmonized and quality-controlled datasets provided the foundation for biomarker discovery using the HMS platform.

Cohort 1 analysis

To eliminate processing and technical batch effects across datasets generated from different laboratories and platforms, we first applied a batch-corrected Bayesian method [2] to co-normalize the seven datasets in Cohort 1 into two data pools: control and diseased (KD and vasculitis). Parameters were estimated from the control samples and then applied to the diseased samples to ensure consistent scaling across datasets. Following normalization, we used the Limma package [4] for univariate gene-level analysis within each of the seven individual discovery datasets. For KD datasets, we compared acute KD samples to FC samples; for vasculitis datasets, we compared vasculitis samples to normal controls. Differential statistics were calculated based on both fold change and Bonferroni-adjusted *p*-values to assess gene-level significance, which contributed to the gene

© 2026 Kuo H et al. *JAMA Network Open*.

weighting for KD and vasculitis in the subsequent analysis. We then used Pathway Analysis (IPA) on the identified dataset genes and pathways perturbations, keeping pathways with more than 10 genes or less than 300 genes in pathway-gene mapping. We followed the weight methodology established by Xiao et al. (2014) to develop a novel significance score for gene selection and ranking [3]. To estimate variation and detect the pathway activity changes in a sample population in a supervised approach, a Pathway Analysis Score (PAS) of each pathway was calculated as the weighted sum of the normalized expression of each gene on the pathway divided by the number of genes in the pathway set:

$$PAS_{jn} = \frac{\sum_{i=1}^{m_j} x_{in} \times \sqrt{|\log_2 F_i|^2 + |-\log_{10} P_i|^2}}{m_j}$$

Where $x_i = \{x_{i1}, \dots, x_{in}\}$ is normalized gene expression profile for gene i and sample n, m_j is the number of genes in pathway j, F_i and P_i indicate fold change and adjusted *p* value for gene i, respectively.

Our approach integrates both statistical significance (*p*-values) and biological relevance (fold changes) for optimal identification of differentially expressed (DE) genes in KD diagnostics. This dual consideration is essential for distinguishing true KD from febrile conditions (FCs) with overlapping clinical presentations. The PAS calculates a weighted sum of normalized differential gene expression within biological pathways, with weights that combine fold change and *p*-value metrics. The squared transformation of log fold changes in our equation normalizes magnitude regardless of direction, emphasizes genes with larger expression differences, and creates a scale-compatible metric that can be meaningfully combined with -$\log_{10}$ transformed *p*-values—a standard approach that converts significance to a positive scale where higher values indicate greater significance.
For each of the seven individual datasets in Cohort 1, pathway variation activity analysis was applied to build a PVA score matrix, and on each PVA score matrix, a Mann-Whitney U test was employed to screen KD and vasculitis DE pathway activity for four KD datasets and three vasculitis datasets. The final pathway signatures are defined as the intersection of KD DE pathways and vasculitis DE pathways representing:

$$Pathway_{signatures} = \left\{\bigcap_{i=1}^{n} P_{KD\ i}\right\} \cap \left\{\bigcap_{j=1}^{m} P_{vasculitis\ j}\right\}$$

Where $P_{KD\ i} = \{pathway \mid p\ value < 0.05\}$ and i is KD dataset i; $P_{vasculitis\ j} = \{pathway \mid p\ value < 0.05\}$ and j is vasculitis dataset j.

This resulted in 13 overlapping pathways: Dendritic Cell Maturation, Cdc42 Signaling, Role of Macrophages, Fibroblasts and Endothelial Cells in Rheumatoid Arthritis, CD28 Signaling in T Helper Cells, IGF-1 Signaling, Role of NFAT in Regulation of the Immune Response, Arachidonic Acid Metabolism, Protein Kinase C theta Signaling in T Lymphocytes, NF-κB signaling, Hepatic Fibrosis / Hepatic Stellate Cell Activation, Colorectal Cancer Metastasis Signaling, Molecular Mechanisms of Cancer, Fc Epsilon RI Signaling (eTable 2). The final gene meta-signatures are screened using criteria: gene signatures must belong to one or more pathway signatures; gene signatures should have significantly different univariate statistics of $|\log_2 fold\ change| > \log_2 1.2$ and $p\ value < 0.05$ in at least one of seven screened datasets.
To ensure clinical translatability of the biomarker candidates, the meta-signature was then filtered through human biofluid proteome databases to prioritize those with known detectability in blood. These included known serum- and urine-detectable proteins obtained from various sources, including the HUPO plasma Proteome Project, a non-redundant list from the plasma Proteome Institute, the MAPU Proteome database, and the Urinary Exosome database, narrowing to 40 candidate genes. In addition, a search of the literature through NCBI identified additional genes differentially expressed between KD and FC but not captured in our initial data-driven screen.

Cohort 2 analysis

For Cohort 2, we combined two microarray datasets and one bulk RNA-seq dataset to enhance the statistical power for identifying KD mRNA biomarkers, including the acute KD and FC samples. Recognizing that combining data from different platforms and studies introduces batch effects, which can confound downstream analyses, we applied a batch-corrected Bayesian normalization method — the same approach used for Cohort 1 — to co-normalize the Cohort 2 data. This method adjusts for technical variation while preserving biological signals. We decided to focus on identifying and constructing the two-gene signature panel using the Cohort 2 data analysis.

© 2026 Kuo H et al. *JAMA Network Open*.

This decision was driven by a strong emphasis on eventual clinical application: minimizing complexity, maximizing assay robustness, and enhancing the feasibility of broad clinical adoption through a cost-effective, simple qPCR assay. The use of a direct gene expression ratio rather than relying on a reference gene in future clinical assays further strengthens the assay's technical reliability and is consistent with best practices in translational molecular diagnostics. The following steps were taken for the cohort data analysis:

(1) Cohort 2 transcriptomic data analysis workflow: We followed a structured, platform-appropriate workflow for transcriptomic data analysis. Raw expression values were normalized for microarray datasets, and differential expression analysis was performed using the LIMMA package [4]. This yielded $\log_2$ fold-change ($\log_2$FC) values and corresponding p-values for each gene. For the RNA-seq dataset, raw read counts were normalized, and differential expression analysis was conducted using the DESeq2 package [5], similarly generating $\log_2$FC values and *p*-values.

(2) Following platform-specific analyses, we conducted a meta-analysis [6] to integrate results across datasets: For *p*-values, Fisher's method was used to combine statistical significance across datasets;

$$X_{2k}^2 \sim -2\sum_{j=1}^{k} log(p_j)$$

For fold-changes, an inverse-variance weighted average was calculated, ensuring that more precise (lower variance) estimates contributed more heavily to the combined effect size.

$$f_{meta} = \frac{\sum_{j=1}^{n} f_j w_j}{\sum_{j=1}^{n} w_j}, \quad w_j = \frac{1}{var(f_j)}$$

This workflow enabled harmonization of findings across heterogeneous data types (microarray and RNA-seq) and enhanced the robustness of biomarker discovery.

(3) Feature Interaction for two-gene ratios: The two-gene ratio approach was designed to capture important biological interactions that might not be evident when evaluating each gene individually. Specifically, calculating the expression ratio between two genes allows us to model situations where disease-relevant changes are driven more by the *relative* expression of gene pairs rather than by *absolute* gene expression levels. This method has two key advantages: (1) It emphasizes gene-gene relationships that could reflect underlying regulatory mechanisms or pathway dynamics. (2) It improves robustness to batch effects and normalization differences across datasets, because ratios inherently normalize shared technical variation.

(4) The meta-metric for each two-gene ratio *x* is calculated as:

$$\underset{x}{\operatorname{argmax}}\left(\sqrt{\left(\frac{\sum_{j=1}^{n} w_j \times \log_2 F_{jx}}{\sum_{j=1}^{n} w_j}\right)^2 + \left(\frac{\sum_{j=1}^{n} w_j \times \left(-\log_{10} P_{jx}\right)}{\sum_{j=1}^{n} w_j}\right)^2}\right)$$

Fx represents the $\log_2$ fold-change of the 2-gene ratio between cases and controls. **Px** represents the negative $\log_{10}$-transformed *p*-value obtained from the statistical comparison of the 2-gene ratio between cases and controls. *x* indexes the specific 2-gene ratio under consideration.

This scoring system ensures that both the *magnitude* of difference (effect size) and the *statistical significance* are integrated into a single metric. Ratios with large fold-changes and small *p*-values (i.e., highly significant differences) will achieve higher meta-metric scores and are prioritized for downstream analysis.

Cohort 3 analysis

Cohort 3 is a single RNA Seq dataset (E-MTAB-11671) and was combined with Cohort 2 for a batch-corrected Bayesian normalization method — the same approach used for Cohort 1 — to co-normalize the Cohort 2+3 data. This method adjusts for technical variation while preserving biological signals. For validation, the previously identified two-gene signature involving *IFI27* and *MCEMP1* was assessed using the following formula:

$$Ratio_{X,Y} = \log_2(\text{transcript } MCEMP1) - \log_2(\text{transcript } IFI27).$$

KD and FC patient qPCR validation cohort, demographic information, and clinical criteria

The diagnosis of KD is confounded due to multiple infectious and inflammatory diseases in children. For this case-control study we recruited pediatric patients with KD and other diseases with symptoms and duration of fever overlapping those found in KD patients. All patients in Cohorts A, B and E were recruited from four medical centers in Taiwan: Linkou Chang Gung Memorial Hospital, Kaohsiung Chang Gung Memorial Hospital, Taichung

© 2026 Kuo H et al. *JAMA Network Open*.

Veterans General Hospital, and Kaohsiung Veterans General Hospital. Samples for cohorts A and B were collected between June 2012 and November 2021. The additional samples collected for Cohort E were collected between November 2017 and March 2023. Cohort C patients were recruited from Children's Hospital Fudan University collected between November 2022 to June 2023. Informed consent was obtained from the parents or guardians of all patients in this study. The studies were reviewed and approved by the Internal Review Boards of Chang Gung Memorial Hospital and Children's Hospital Fudan University. All blood samples were anonymized and tested only after clinical assignments were finalized.

All patients recruited for this study were diagnosed with KD according to the American Heart Association (AHA) criteria. Patients were classified as either complete KD — defined as having a fever lasting more than 5 days and at least four out of five principal clinical features (bilateral non-suppurative conjunctivitis, oral mucosal erythema or fissured lips, cervical lymphadenopathy, dysmorphic skin rash, and edema or erythema of the extremities) — or incomplete KD, in which patients exhibited fewer than four principal clinical features but still fulfilled the AHA diagnostic guidelines for incomplete KD.[7] Each patient was evaluated independently by one of two expert KD physicians, with the final diagnosis based on consensus. Blood samples were obtained from KD patients within 24 hours prior to IVIG therapy.

Dilated coronary arteries and aneurysms are diagnosed by the treating physician using the coronary z-score, which measures the coronary artery internal diameter normalized to the patient body surface area. The z-scores were classified into three categories: No coronary involvement (z-score < 2.0), dilation only (z-score 2.0 to 2.5), and aneurysm (z-score > 2.5).

Febrile control patients were those with a continuous fever of two or more days referred to a KD specialist, admitted and requiring blood tests with an alternative diagnosis (eTable 5). Since there is no gold standard for diagnosing bacterial and viral diseases, each sample was independently classified by three experienced physicians given access to available electronic Case Report Form (eCRF) information. Each physician assigned one of the following five categories to each sample: Viral infection, bacterial infection, undetermined infection type (either viral or bacterial), non-infectious or unknown. The final classification was based on the majority agreement when at least two physicians agreed to on the classification of each sample. Patients were excluded if they were previously treated with antibiotics or were considered in recovery. In addition, FC were matched in age to the KD patients and were excluded if over 8 years of age.

Biomarker validation using RT-qPCR

Cohorts A, B, C, D, and E were tested using the RNA extracted from PBMCs. Whole blood was also used in cohort E (CAP/CLIA validation). Whole blood samples were first centrifuged at 3000 RPM for 10 minutes, and the plasma was removed. The red blood cells were then lysed using RBC lysis buffer, and PBMCs were pelleted by centrifugation. Total RNA was isolated from the cell pellet, and RNA was extracted using the Ambion AM1560 kit (ThermoFisher Scientific, Waltham, MA, USA). Total mRNA from all samples was reverse-transcribed into cDNA using the TaqMan MicroRNA Reverse Transcription Kit (Thermo Fisher Scientific, Waltham, MA, USA), and the cDNA was stored at -80°C. cDNA stock was diluted 20X, and 2 µL was used for each qRT-PCR reaction. Primers for qPCR were predesigned from IDTdna and validated by determining linearity and reaction efficiency and examining melt curves. For testing of Cohorts A, B, C and D, RT-qPCR was performed in duplicate using 2xiTaq Universal SYBR Green Supermix (BioRad, Hercules, CA, USA) with Cohorts A and B run on a CFX96 thermocycler (BioRad, Hercules, CA, USA) and Cohorts C and D run on an ABI 7500 (ThermoFisher Scientific, Waltham, MA, USA). For the CAP-validated validation, qRT-PCR was performed in duplicate using PrimeTime One-Step RT-qPCR Mast Mix (IDTDNA, Coralville, IA, USA) on a QuantStudio5 Dx thermocycler (ThermoFisher Scientific, Waltham, MA, USA). The assays were further validated through extensive analytical methods during LDT development.[8,9] GAPDH normalization is used to verify RNA integrity, RT efficiency, and assay performance. Samples with failed or unstable GAPDH amplification can be flagged and excluded. This is essential for CLIA/CAP analytical validity.

Samples failing initial qPCR quality-control criteria (no amplification or excessive replicate variability) were re-extracted and re-assayed. Only samples meeting predefined QC thresholds were included in the final analytic dataset. No samples were excluded due to persistent assay failure.

Each cohort was tested, and KD vs FC were compared using the two-gene combinations by intensity ratios using the equation:

$$\text{KD score} = \Delta\text{Ct}(\mathit{IFI27}) - \Delta\text{Ct}(\mathit{MCEMP1})$$

© 2026 Kuo H et al. *JAMA Network Open*.

The single cutoff in the binary model was determined using the Youden index. Sensitivity, specificity, positive predictive value (PPV), and negative predictive value (NPV) were computed based on the optimal cutoff score with 95% confidence intervals for all metrics. A two-threshold model was developed by adjusting the threshold to minimize the number of indeterminate samples while ensuring a PPV and NPV greater than 0.95. The sensitivity, specificity, PPV, and NPV were computed based on the high and low-scored samples. The indeterminate samples were excluded as those patients should be recommended for monitoring, follow-up and retesting. The student's T-test compared the KD patients' analyte Ct values with the febrile controls. All statistics were analyzed via R software, version 4.3 (R Foundation for Statistical Computing). Two-sided *p* values were determined, and *p* value <0.05 are considered significant.

Statistical Analysis

All statistical analyses were performed in R software, version 4.3 (R Foundation for Statistical Computing), with preprocessing steps and methods as defined in each section above. Sample size was determined by available prospectively collected specimens following completion of transcriptomic discovery. The resulting cohort size exceeds recommended minimums for stable AUC estimation in diagnostic accuracy studies and enabled prespecified subgroup analyses without model re-optimization.

Biomarker identification through multicohort gene prioritization

To systematically identify a robust gene signature capable of distinguishing KD from other pediatric febrile illnesses, we developed and implemented the Hyperflex-Meta-Signature (HMS) platform—a modular, high-throughput bioinformatics pipeline that integrates statistical rigor, pathway-based biological relevance, and cross-cohort consistency. The analysis encompassed 11 publicly available transcriptomic datasets categorized into three cohorts (eTable 1). Within the discovery cohort (Cohort 1), which included KD, vasculitis, and control samples, differentially expressed genes (DEGs) were first identified using a batch-corrected normalization approach followed by univariate statistical testing.

To incorporate biological context, we computed pathway activity variation scores across datasets and applied Ingenuity Pathway Analysis (IPA) to identify signaling cascades enriched in KD and vasculitis samples. This yielded 13 conserved immune and inflammatory pathways (eFigure 1, eTable 2), from which 82 candidate genes were extracted. To ensure clinical feasibility and blood-based detectability, we filtered these candidates against plasma- and exosomal-proteomic databases, yielding a narrowed list of 40 genes. An additional 14 genes were incorporated based on previous literature linking them to KD or systemic inflammation [10,11], generating a list of 54 gene candidates for further evaluation (eTable 3).

We next applied our cross-platform meta-analysis framework to Cohort 2, which included both microarray and RNA-seq datasets. All possible two-gene combinations derived from the 54 candidates were systematically evaluated to identify the most diagnostically informative pair (eFigure 1B). Each combination was ranked based on a composite meta-metric that integrated effect size (log_2 fold-change) and statistical significance (meta-analyzed p-values), weighted by variance and cohort concordance (see Methods). Among all combinations, the pairing of IFI27 and MCEMP1 emerged as the top-performing signature, achieving an absolute log_2 fold-change of 4.95 and a meta-analyzed *p*-value < 0.0001 (eTable 4). These two genes demonstrated opposing regulation in KD: MCEMP1 was consistently upregulated, while IFI27 was markedly downregulated in KD relative to febrile controls across all training datasets.

Importantly, the selection of IFI27 and MCEMP1 was derived entirely from Cohorts 1 and 2. These genes were not pre-specified or tuned using downstream qPCR or independent validation cohorts, thereby mitigating selection bias and preventing circular validation, limitations sometimes encountered in biomarker development. This biologically coherent and statistically robust two-gene signature served as the foundation for subsequent RT-qPCR assay development and clinical testing.

Index test blinding statement

Laboratory personnel were blinded to clinical diagnosis, and clinical adjudicators were blinded to qPCR results."

Verification of a two-gene signature: Ensuring statistical independence and translational robustness.

To further assess the robustness and reproducibility of the candidate genes, we evaluated their expression independently across four datasets (eFigure 2) from Cohorts 2 and 3, which included both microarray (Microarray 1 and Microarray 2) and RNA-seq platforms (RNAseq 1 and RNAseq 2, eTable 1). Consistent differential expression was observed across all datasets: IFI27 was significantly downregulated in KD relative to febrile

© 2026 Kuo H et al. *JAMA Network Open*.

controls (p < 0.0001 in all datasets), while MCEMP1 was significantly upregulated (p < 0.01 in all datasets; p < 0.0001 in three).
To quantify diagnostic performance, we computed a KD score using the expression ratio of IFI27 to MCEMP1 across the same transcriptomic datasets. This metric consistently yielded significantly higher scores in KD patients compared to febrile controls (p < 0.0001 across all datasets; eFigure 3). Receiver operating characteristic (ROC) analysis demonstrated moderate to high classification performance, with area under the curve (AUC) values of 0.81 and 0.84 for the two microarray datasets and 0.96 and 0.75 for the two RNA-seq datasets, respectively. These results highlight both the strength and the variability of transcriptomic classification across platforms, acknowledging the greater technical noise and signal compression inherent to microarrays and the potential for variability in RNA-seq quantification at the low expression range.
Together, these findings support the reproducibility of the IFI27/MCEMP1 gene signature across independent datasets and molecular profiling technologies. This cross-platform consistency formed the analytical foundation for its subsequent translation into a qPCR-based clinical assay.

RT-qPCR Analytical Validation

All analytical validation was performed as part of a CAP-accredited, CLIA-certified laboratory-developed test (LDT). The analytical validation is performed to ensure the assay is consistent and reproducible meeting predetermined specifications [8,9]. The analytical validation consists of Accuracy, Linearity, Analytical Sensitivity, Precision, and Analytical Specificity. The primer and probe sequences used in this validation are indicated in eFigure 4. In addition, the possibility of common blood interferants and the robustness to determine storage parameters were tested. All analytical validation studies were performed on blood from healthy adults.

The analytical validation studies demonstrated the assay's high accuracy for *IFI27* and *MCEMP1*, with R^2 values> 0.997, indicating accurate detection and quantification of the targeted genes (eFigure 4). Linearity (eFigure 6) and analytical sensitivity (eTable 8) testing indicated robust total RNA input from 1.562 to 100 ng. Moreover, evaluation of precision demonstrated minimal variation in results over a 20-day period (eFigure 7), while specificity testing confirmed the detection of a single correctly sized qPCR product for each gene, unaffected by genomic DNA contamination (eFigure 8 and eTables 9-11). Additional studies ensured that common blood interferents did not affect the assay performance and indicated sample stability through day 6 when stored at optimal conditions (eTables 12-13).

Accuracy

Accuracy or trueness is the extent to which an assay agrees with a true or a reference value. A recovery study tests whether the assay can measure an analyte when the input of that analyte is known. A 10-fold dilution series of known concentrations of synthesized double-stranded DNA fragments for the gene region amplified by the primers was used as a template for the reaction. The template ranged from 10,000,000 copies to 10 copies. The Ct values for both genes, plotted by the # of copies, fit with a logarithmic trendline (eFigure 5). The trendline for *IFI27* has an R^2 of 0.9995, and *MCEMP1* has an R^2 value of 0.9967. An R^2 value >0.98 indicates the primer/probe accurately detects the correct nucleotide sequence.

Linearity

The reportable range is the range of Ct values at which the assay can reliably be quantified and is defined by the range at which the results are linear [8]. The linear range is determined by dilutions of synthesized double-stranded DNA fragments as done for accuracy. In addition, the linear range is confirmed by a standard curve of total RNA extracted from blood samples in which the analyte concentration is unknown. Since the qPCR diagnostic assays are based on relative concentration, the absolute RNA concentration is unnecessary. RNA was extracted from three whole blood samples. Total RNA from each sample was diluted to eight inputs for RT-qPCR for each gene to determine a standard curve based on total RNA (eFigure 6). The R^2 value of the linear standard curve should be ≥0.98 for each assay. Data points were excluded if there is no amplification or if the standard deviation is > 0.6. The linear range of the Ct values for *IFI27* is 13.76 to 36.39, and *MCEMP1* is 14.62 to 37.29 (eFigure 6).

In addition, the standard curve results are used to define PCR efficiency. PCR efficiency defines how close the observed assay is to an actual doubling of the qPCR product over successive cycles [8]. An acceptable efficiency of a qPCR assay is between 90% and 110%. The PCR efficiency is calculated from the average of 3

blood samples and the DNA template. The PCR efficiency of *IFI27* is 102.39% and for *MCEMP1* is 100.34% (eFigure 4).

Analytical Sensitivity (LLOQ)

The analytical Sensitivity or lower limit of quantification (LLOQ) is the lowest amount of the target analyte that can be reliably quantified [8,9]. The LLOQ is defined as the lowest RNA input with a standard deviation ≤0.5 and %CV ≤20% in five samples. RNA was extracted from five whole blood samples, and total RNA was diluted to an RNA input of the lowest linear total RNA concentration defined by the reportable range and 1/2X dilutions lower and 2X higher (eTable 8). LLOQ of the *IFI27* assay is 1.562 ng input RNA and a Ct of 35.70. The LLOQ of the *MCEMP1* assay is 196 pg of total RNA input and a Ct of 36.98.

Analytical Precision

Analytical precision measures the variability of independent measurement under specific conditions [8,9]. This is done by testing aliquots of the same sample 20 times over 20 non-consecutive days with an expected %CV < 20%. Whole blood from one individual was aliquoted into 20 1.5 mL samples and frozen. A single sample each day for 20 days was thawed, RNA extracted and tested by qPCR assayed on three concentrations of total RNA (low, mid, and high) as determined by the linearity curve. The precision of the assay was tested on a single machine with two operators and illustrates the precision of the assay with %CV below 20% for all three total RNA concentrations (eFigure 7). In addition, the variability of the Ct range is defined by the difference between the greatest and least Ct values, which is 1.21 for *IFI27* and 1.70 for *MCEMP1*.

Analytical Specificity

Analytical specificity ensures that the assay is specific for the target gene without being affected by related genes or interfering substances. Primer-Blast was used to check the specificity of the primers and probe to the intended target. Primer-Blast is a computer program that checks a primer's sequence against the entire human genome, identifying targets with a significant number of matches to the primers. Based on our Primer Blast analysis, only *IFI27* matches the primers 100%. No other genes were identified as unintended targets. The *MCEMP1* primer and probe sequences match the *MCEMP1* sequence 100%. While *MCEMP1* primer 2 has 4 of 19 matching nucleotides for the unintended target SFT2D2, neither primer 1 nor the probe have similarity to this gene, preventing any possibility of amplifying an unintended target in the human genome.

A second method to verify analytical specificity is through examining the PCR product on an agarose gel. The PCR product is expected to have a single band of the correct size. Additional bands or the wrong size band would indicate unintended target amplification. PCR products from 3 blood samples were run on an agarose gel to test for the expected amplicon size. Each PCR product has a single band of the expected size of 118 bp for *IFI27* and 114 bp for *MCEMP1*, confirming that the primers/probe assays are highly specific to their respective genes (eFigure 8).

Another possible source of analytical specificity error is contamination of the assay with the target gene or amplified product. The assay for each gene, *IFI27* and *MCEMP1*, was run 20 times without template, alongside a test sample with RNA, to test for contamination or cross-reactivity of the primers and probes. All 20 no template controls (NTCs) had undetermined Ct values, confirming that the assay does not generate Ct values when a NTC (i.e., a blank) is processed.

Genomic DNA is another source of contamination that can lead to errors in analytical specificity. To test for this, two commercially procured total human gDNA samples, from male and female donors, were tested in 5 replicates, each at 10 and 100 ng per reaction. A regular RNA sample was run with each gDNA sample to verify the assay function. No Ct value for IFI27 was generated when gDNA was used as input, suggesting no interference from genomic DNA (eTable 6). *MCEMP1* though indicated a robust signal with a Ct value <30 for each gDNA sample at both tested concentrations suggesting this assay is not specific to mRNA but may also detect *MCEMP1* genomic DNA.

The *MCEMP1* primers were designed to span an intron between exon 6 and exon 7. The intron though is relatively short at 114 bps, allowing the theoretical possibility of genomic DNA amplification. The genomic DNA would generate a 228 bp amplicon whereas the cDNA (mRNA-derived) amplicon is only 114 and has a distinct melting profile shifted to the right indicating a higher molecular weight (eFigure 9). The RNA extraction protocol

© 2026 Kuo H et al. *JAMA Network Open*.

utilizes a DNase to remove genomic DNA present in the sample. In our experiments, we consistently observed a single band at 114 bp (eFigure 8) and a single melting curve peak, without evidence of larger amplicons.

To demonstrate that the DNA is degraded in the column during DNAse digestion, the same two gDNA samples, in place of blood, were loaded onto the column, and extracted following the DNAse and wash protocols. Each sample was tested with and without the DNase at both 15 min, incubated at room temperature (RT), or 30 min, incubated at 37°C. The elution was then assayed for *MCEMP1* using RT-qPCR (eTable 10). There was no detectable DNA in the female sample treated with DNAse for 15 min RT, and the male sample was detected, though it had a higher Ct value (31.8) than the non-DNase treated sample (25.53). Increasing the temperature of the DNase digest improves the DNA digestion as the DNA is only detected in two of the five male DNA replicates, but the Ct value is at 38.22, which is above the determined LLOQ for *MCEMP1* (eTable 10).

To further demonstrate that the genomic DNA does not affect the *MCEMP1* RT-qPCR assay, the extraction protocol was carried out on three whole blood samples with and without DNase at either 15 min at room temperature or 30 min at 37°C. The mean Ct values for each treatment were compared using a Paired T-test (eTable 11). Samples treated with DNase at RT and 37°C show no difference in Ct values ($P = 0.722$). In addition, a comparison of samples treated with and without DNase indicates no difference at room temperature ($P=0.196$) or at 37°C ($P=0.870$). Together, these data demonstrate that though this *MCEMP1* primer/probe assay can detect genomic DNA, the genomic DNA that may be present in the sample does not affect the quantification of *MCEMP1* mRNA.

Interfering Substances

Interfering substances refers to any compound capable of affecting the measurement of the analyte by interfering with nucleotide amplification. Interfering substances can be sourced endogenously in the sample or from an exogenous source such as medications taken. A list of possible interfering substances common in pediatric blood and their recommended testing concentrations was identified. These possible interfering substances were tested in groups of five interferents spiked into whole blood at the concentrations listed in eTable 12 and compared with the same sample spiked with diluent. Each group of interferents was tested in blood from three individuals, and each sample assayed in duplicate for qPCR. Three groups were tested simultaneously with a control sample (A/B/C Ctrl or D/E/F Ctrl). The interferent group is considered to affect qPCR if a paired T-test shows a $P < 0.05$ and the average absolute delta Ct between an interferent spiked sample and a control diluent sample is > 0.5 Ct. The measurement of IFI27 and *MCEMP1* are not affected by any interferent group eTable 13.

Sample Storage Robustness

Robustness methods test the effect on the assay of less-than-ideal situations in the assay. The samples in this analytical validation used samples that were frozen shortly after the blood was drawn and stored at -80°C and shipped on dry ice. While this is ideal, it is unlikely to occur clinically. Samples in the clinic will likely be stored at 4°C and shipped on ice. To test the effect storage at 4°C and at RT may have on the assay, whole blood samples were collected from three individuals and aliquoted into 1.5 mL aliquots. One aliquot was frozen at -80°C on day 0 while the others were stored at 4°C or RT. Aliquots from both temperature groups were frozen on day 1, day 2, day 4 and day 6 after blood draw. Each sample was thawed, RNA extracted and run on qPCR. Samples are considered significantly different from day zero if the average difference in Ct value is greater than the range in Ct values seen in the mid concentration (8ng input) of the whole blood precision assay which for *IFI27* is 0.77 and *MCEMP1* is 1.69 (eFigure 3). The results indicate that the measurement of *IFI27* and *MCEMP1* in blood is not affected when the sample is stored at 4°C for up to six days or at RT for up to one day (eTable 14).

© 2026 Kuo H et al. *JAMA Network Open*.

Supplementary Figures

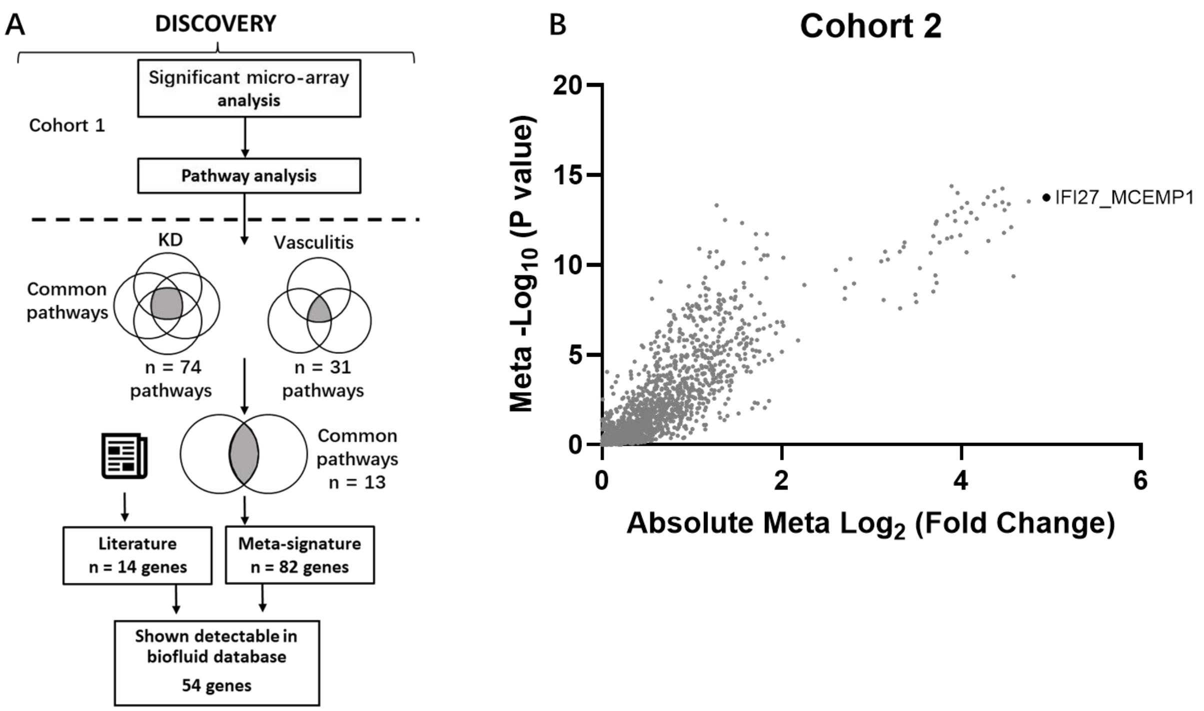

eFigure 1. Discovery and screening of optimized 2-gene pairs for differential expression in Kawasaki Disease.

Identification of candidate genes based on differential expression analysis (A). Screening of candidate 2-gene pairs in Cohort 2 (B). Each gene pair is plotted with the absolute meta $\log_2$ fold-change on the x-axis and the absolute meta $\log_{10}$-transformed *p*-value on the y-axis. This approach highlights gene pairs with both strong differential expression and statistical significance. KD, Kawasaki Disease

© 2026 Kuo H et al. *JAMA Network Open*.

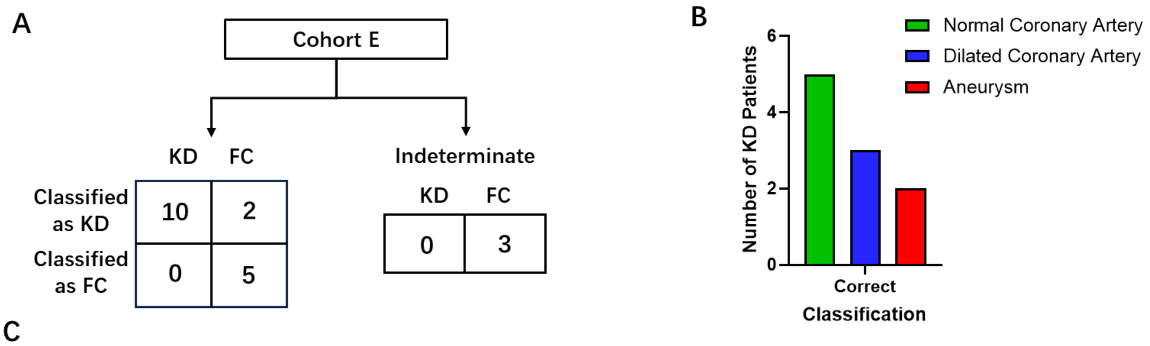

C

Gene Symbol	Assay Sequences		Assay Range		Linearity	Precision	Specificity	RNA stability in whole blood storage
			Low (LLOQ)	High				
IFI27	Primer 1	ACA ACT CCT CCA ATC ACA ACT	Total RNA input (ng)		Standard Curve R2=0.996	≤ 1.06 % CV	Primer pair is unique for IFI27	2 days at RT
	Primer 2	CCT CTG CTC TCA CCT CAT CA	1.562	100				
	Probe	/56-FAM/TC TGG CTC T/ZEN/G CCG TAG TTT TGC C/3IABkFQ/	Ct value LLOQ					
			35.7	13.755				6 days at 4°C
MCEMP1	Primer 1	TCATTTCCCGTCTTCGTTGG	Total RNA input (ng)		Standard Curve R2=0.991	≤ 1.72 % CV	Primer pair is unique for MCEMP1	1 day at RT
	Primer 2	GAGGTGCTGCAGAAAATGC	0.196	100				
	Probe	/56-FAM/TCAATAAAT/Zen/GAGAGGACATTGTGGCAGCC/3IABkFQ/	Ct value LLOQ					
			36.98	14.62				6 days at 4°C

eFigure 2: Clinical and analytical validation for a laboratory developed test.
A 2x2 table of Cohort E (A) classification. Performance of the KD score to correctly classify patients with coronary artery complications in cohort E (B). Summary of the analytical validation (C) for *IFI27* (top) and *MCEMP1* (bottom) FCs, febrile controls; KD, Kawasaki disease; LLOQ, lower limit of quantitation; RT, room temperature.

© 2026 Kuo H et al. *JAMA Network Open*.

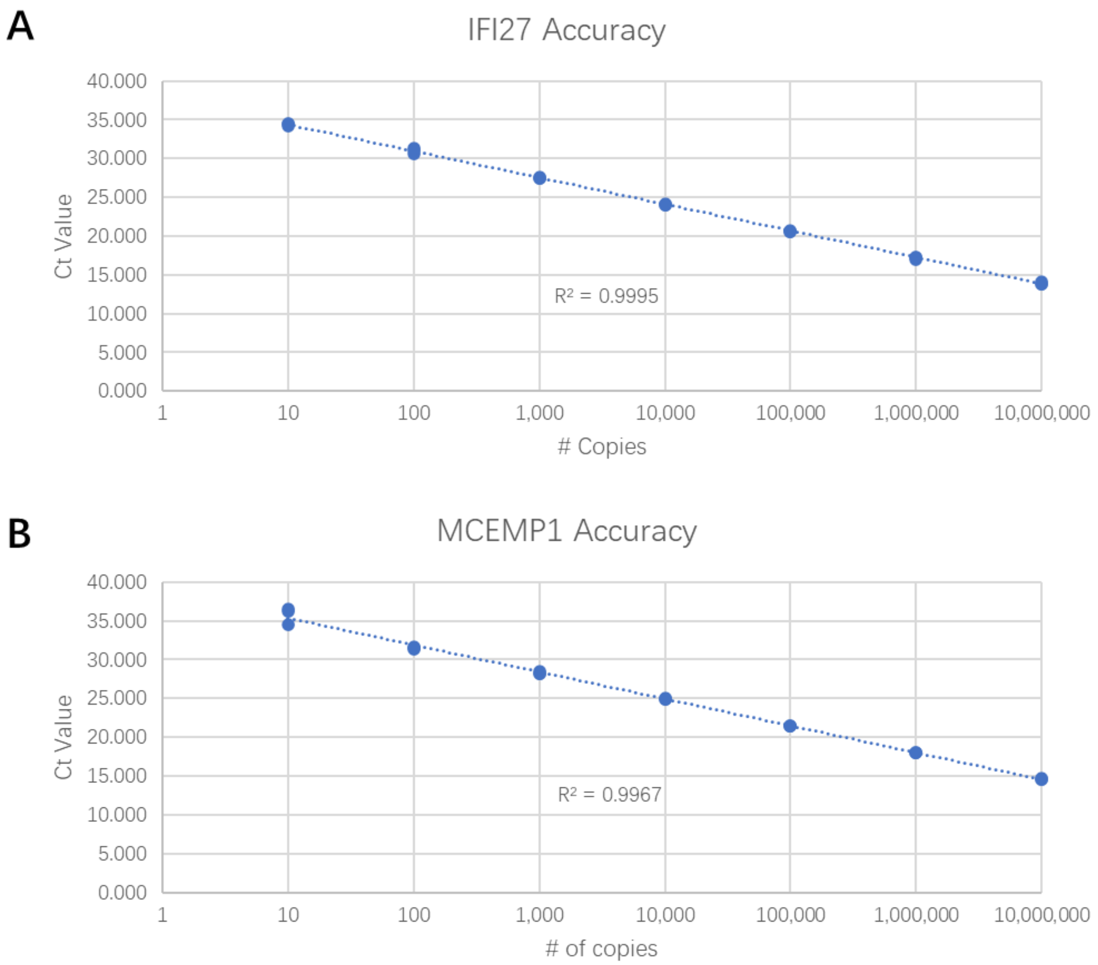

eFigure 3: Accuracy of the *IFI27* and *MCEMP1* assays.

10-fold dilution series of synthesized double stranded DNA gene fragment for *IFI27* (A) and *MCEMP1* (B).

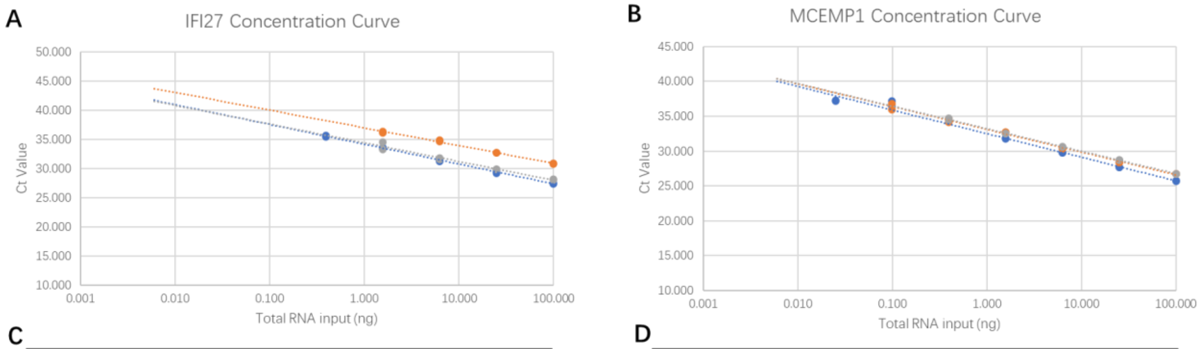

C

Sample	R2	Efficiency		Linearity Range	
				Minimum	Maximum
IFI27 DNA standard	0.999	95.99	Input	10 copies	10,000,000
			Ct	34.507	13.755
Sample 1	0.998	97.394	Input	391 pg	100 ng
			Ct	35.484	27.418
Sample 2	0.996	113.75	Input	1.562 ng	100 ng
			Ct	36.393	30.816
Sample 3	0.99	96.014	Input	6.250 ng	100 ng
			Ct	32.984	29.191
**Combined**	**0.995**	**102.39**	**Input**	**1.562 ng**	**NA**
			**Ct**	**36.393**	**13.755**

D

Sample	R2	Efficiency		Linearity Range	
				Minimum	Maximum
MCEMP1 DNA standard	1.000	97.62	Input	10 copies	10,000,000
			Ct	36.472	14.615
Sample 1	0.988	97.311	Input	25 pg	100 ng
			Ct	37.287	25.649
Sample 2	0.995	102.524	Input	98 pg	100 ng
			Ct	36.839	26.698
Sample 3	0.999	103.908	Input	391 pg	100 ng
			Ct	34.714	26.774
**Combined**	**0.994**	**100.34**	**Input**	**98 pg**	**NA**
			**Ct**	**37.287**	**14.615**

eFigure 4: Linearity of *IFI27* and *MCEMP1* assays.

© 2026 Kuo H et al. *JAMA Network Open*.

Concentration curve of 4-fold dilution series of total RNA from three samples tested for *IFI27* (A and C) and *MCEMP1* (B and D).

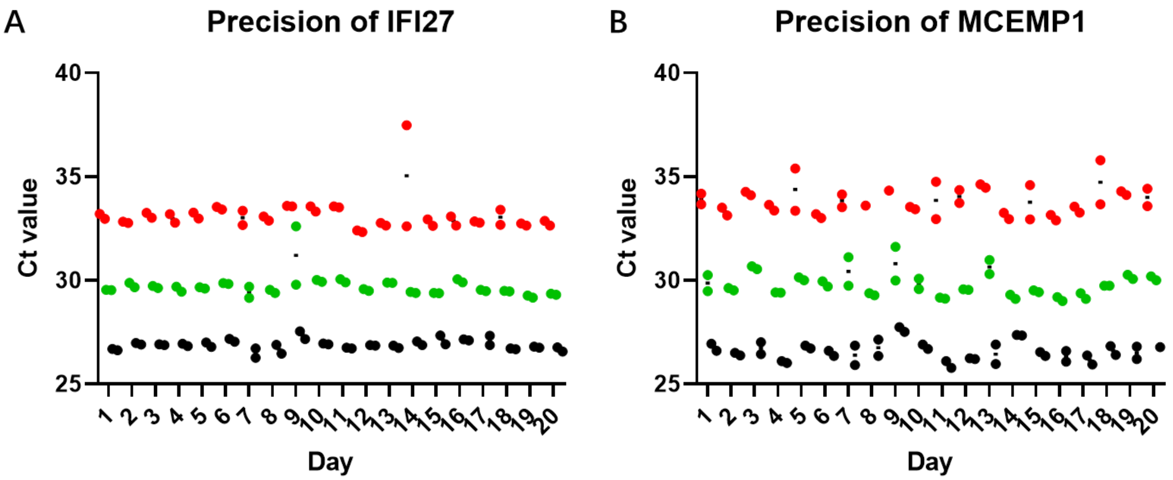

C

Gene	Total RNA	Mean Ct	SD	Inter-Assay CV%	Ct range
IFI27	80 ng	26.89	0.20	0.75%	0.87
	8 ng	29.62	0.23	0.76%	0.77
	0.6 ng	32.99	0.35	1.06%	1.21
MCEMP1	80 ng	26.58	0.39	1.47%	1.69
	8 ng	29.81	0.51	1.72%	1.69
	0.6 ng	33.82	0.48	1.43%	1.70

eFigure 5: *IFI27* and *MCEMP1* have good precision performance.
Ct values of replicates extracted from the same sample over 20 days and assayed on RT-qPCR using *IFI27* (A) and *MCEMP1*(B) assays. Each day tested 80 ng (high total RNA) 8 ng (mid total RNA) and 0.6 ng (low total RNA) and were analyzed by SD and Inter-Assay CV% (C).

© 2026 Kuo H et al. *JAMA Network Open*.

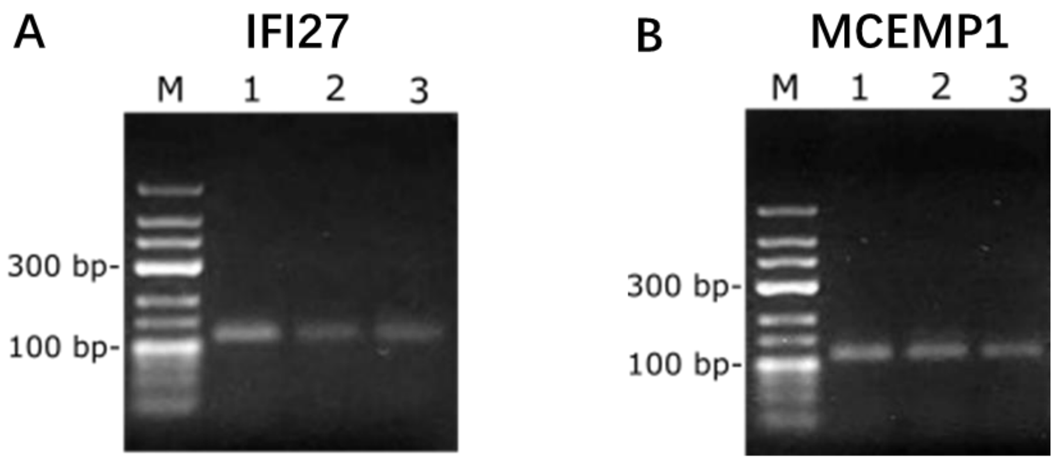

eFigure 6: Assays detect specific RNA for *IFI27* and *MCEMP1*.
Agarose gel stained with SYBR Safe DNA gel stain for three RT-qPCR products indicates a single band around 118 bp for *IFI27* (A) and 114 bp for *MCEMP1* (B).

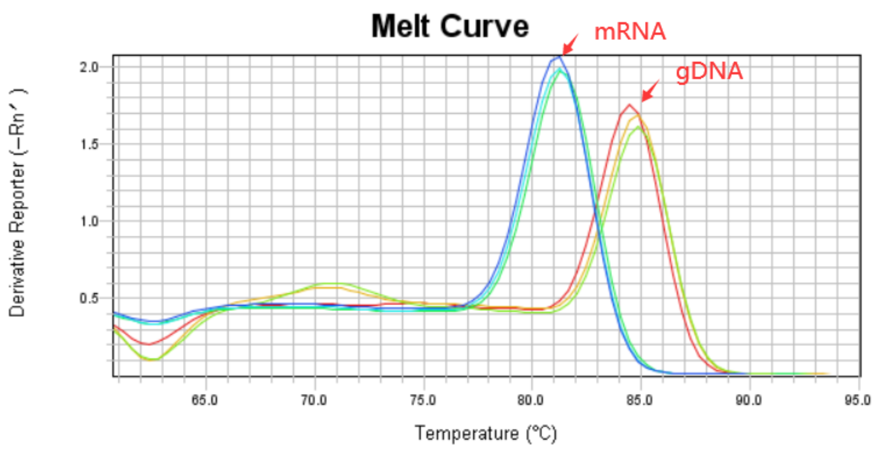

eFigure 7: gDNA of MCEMP1 has a distinct shifted melting temperature.

SYBR green qPCR of gDNA shows a distinct melting temperature to the right of the mRNA.

© 2026 Kuo H et al. *JAMA Network Open*.

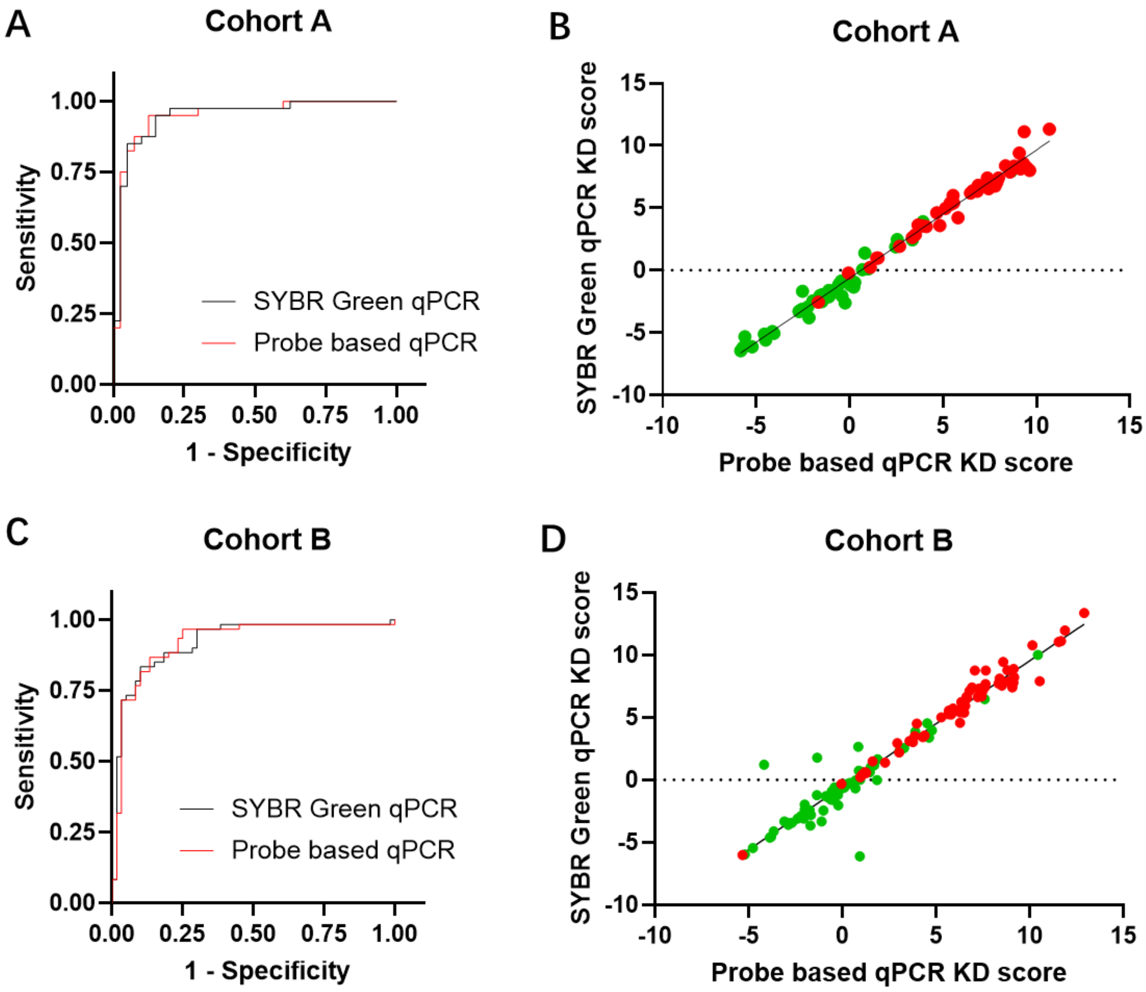

eFigure 8: Portability of RT-qPCR.

The same samples were tested using SYBR green qPCR in our Taiwan lab and Probe based qPCR in our US CAP accredited lab. ROC curves were generated for Cohort A (A) and a portion of Cohort B (C). Linear regression analysis compared the SYBR green performance (Y axis) to the Probe based performance (X axis) for Cohort A (B) and Cohort B (D).

© 2026 Kuo H et al. *JAMA Network Open*.

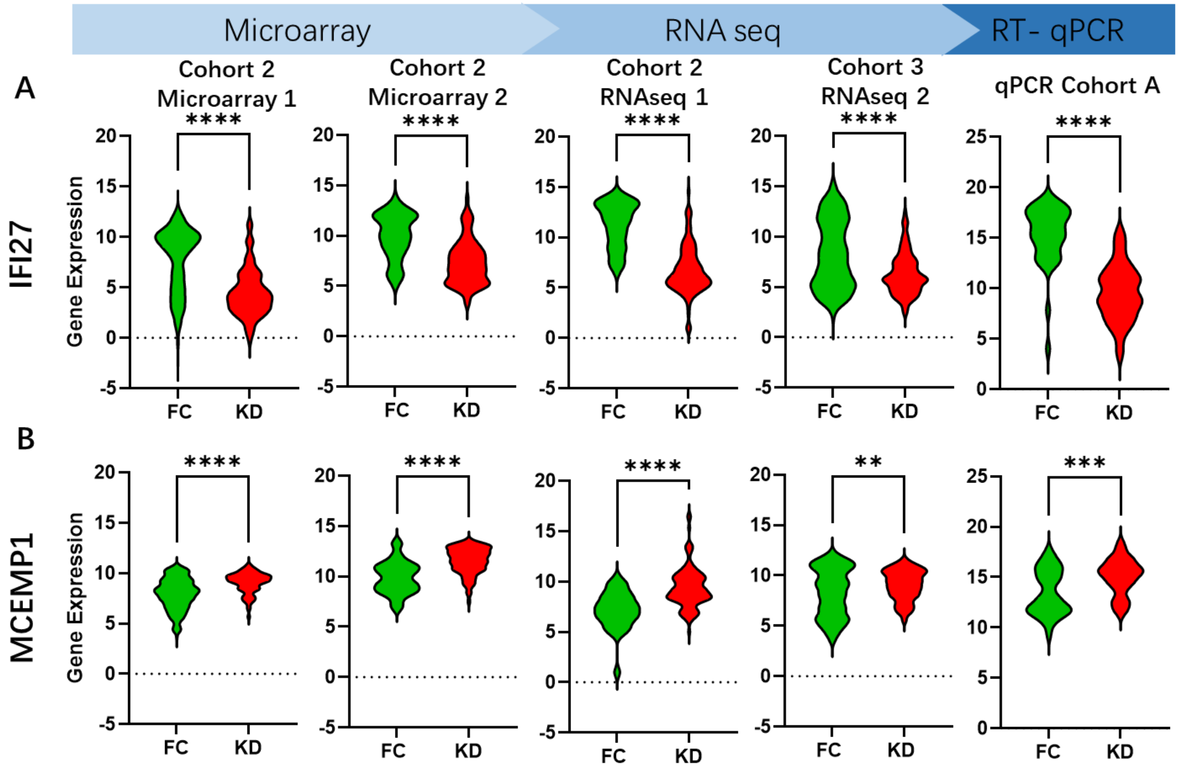

eFigure 9: Individual gene validation transition from Microarray to RNAseq to RT-qPCR.

For each gene, Violin plots for *IFI27* (A) and *MCEMP1* (b) gene expression shown for FC (green) and KD (red) in two Microarray (Cohort 2), two RNAseq (RNAseq 1 from Cohort 2 and RNAseq 2 from Cohort 3) and one qPCR cohort (Cohort A. **p*<0.05, ****p*<0.001 and *****p*<0.0001 (Student T test) FCs, febrile controls; KD, Kawasaki disease.

© 2026 Kuo H et al. *JAMA Network Open*.

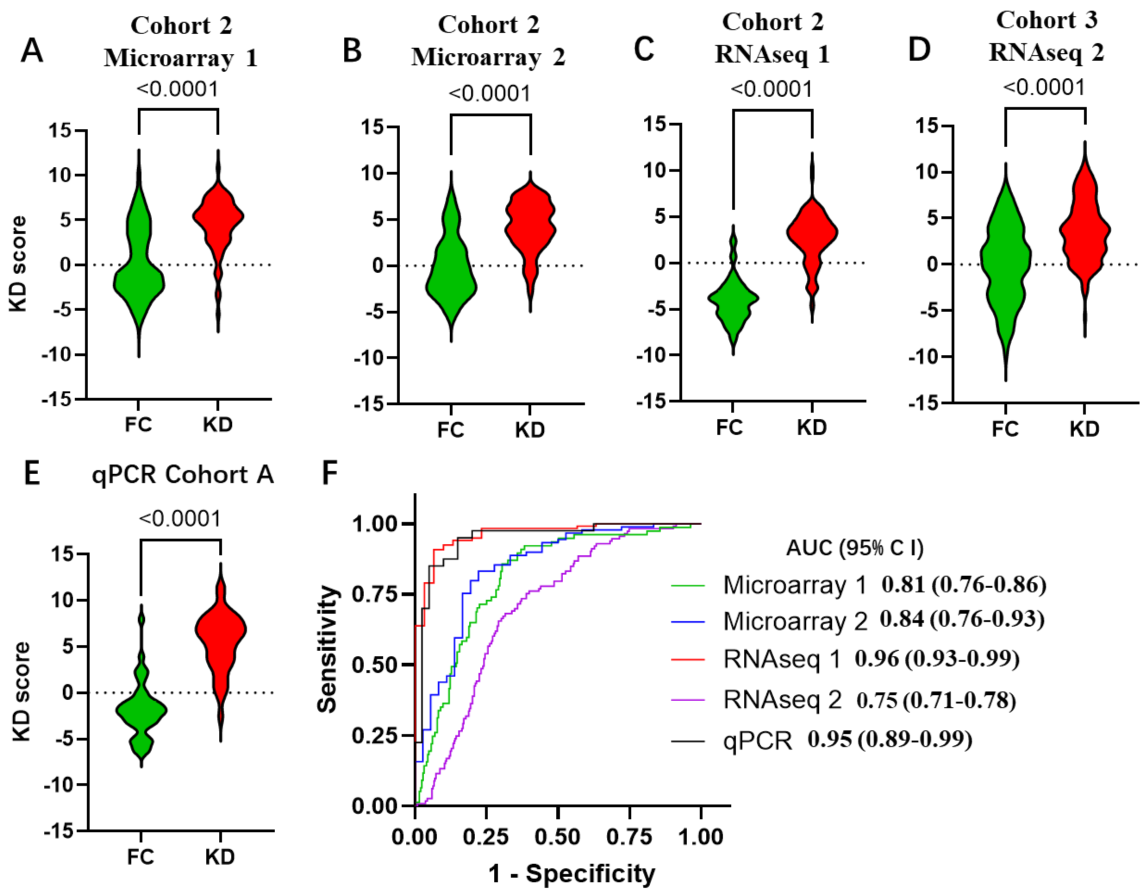

eFigure 10: KD score validation in Microarray, RNAseq and RT-qPCR.
Violin plots of FC and KD for cohorts tested by microarray (A and B: Cohort 2), RNAseq (C: Cohort 2; D: Cohort 3), and qPCR (E: Cohort A). ROC curves were generated for each individual data set (F) with the AUC value and 95% CI. *p* values calculated using Student T test; FCs, febrile controls; KD, Kawasaki disease.

© 2026 Kuo H et al. *JAMA Network Open*.

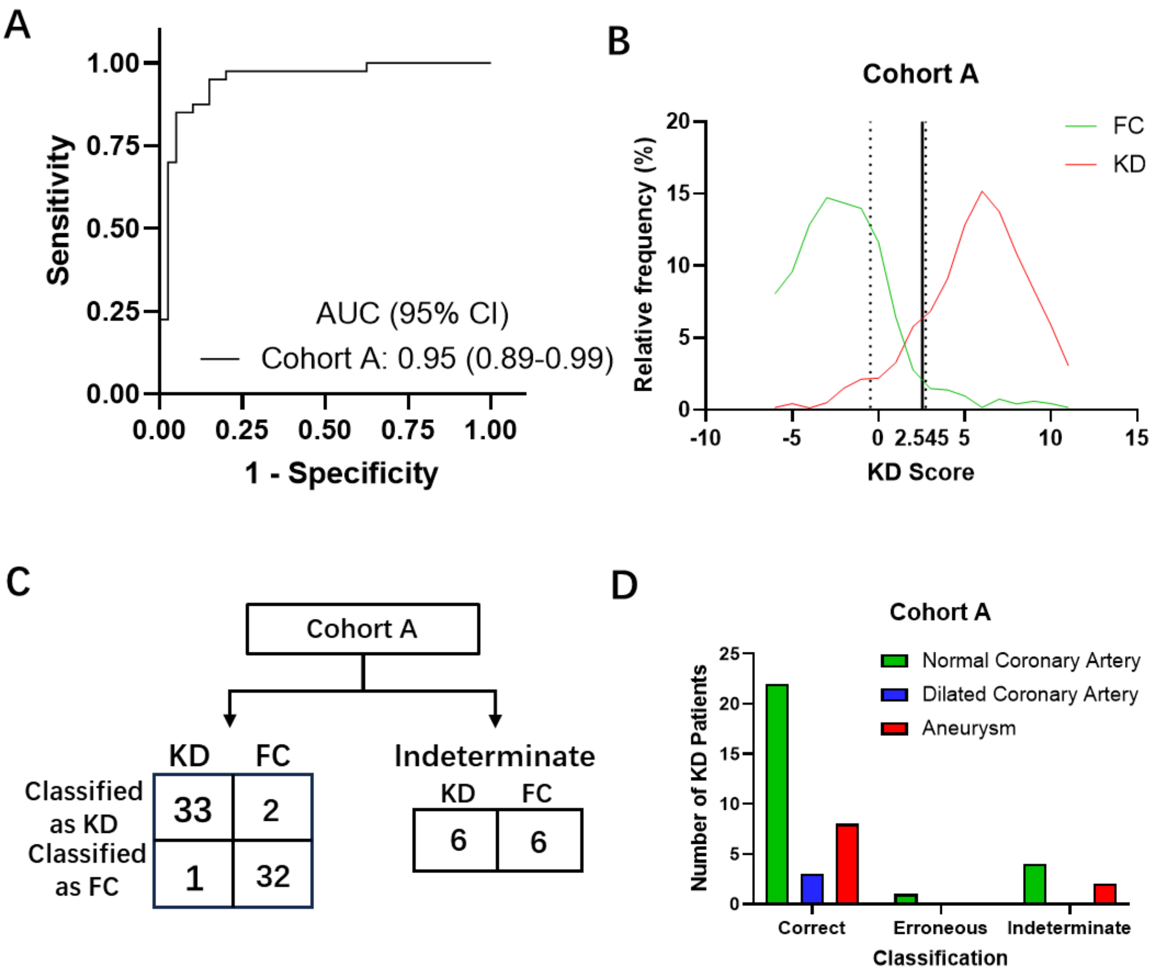

eFigure 11: KD assessment with cohort A and diagnostic threshold determination.

(A). Cohort A KD score used to optimize thresholds for qPCR (B) with a single threshold (solid vertical line) and two-thresholds (dotted vertical lines). A 2x2 table of Cohort A (C), classification. Performance of KD score to correctly classify patients with coronary artery complications in cohort A (d). FCs, febrile controls; KD, Kawasaki disease. The indeterminate range is an intentional and clinically motivated feature of the diagnostic framework, rather than a result of assay failure or missing data. Consistent with our prior publication establishing the KD score framework[12], the diagnostic thresholds were defined to ensure that the low and high score ranges achieved negative predictive values greater than 95% for febrile controls and positive predictive values greater than 95% for Kawasaki disease, respectively. Scores falling between these thresholds represent a biological overlap zone in host inflammatory response, not technical qPCR failure. These samples, therefore, do not require repeat specimen collection or assay repetition; instead, they reflect cases in which immediate molecular classification alone is insufficient for a definitive diagnosis. In Cohort A, this indeterminate range was prospectively defined during threshold optimization, and its prevalence is explicitly reported. Patients with indeterminate scores were not excluded due to assay inadequacy; rather, they were analyzed separately to characterize clinical outcomes. As shown in eFigure 11 and validated across independent cohorts in eFigure 12, patients with indeterminate scores exhibited coronary artery outcomes intermediate between those classified as KD-positive and KD-negative, supporting the biological and clinical relevance of this category. From a clinical perspective, the indeterminate range functions as a safety buffer that prioritizes diagnostic certainty, allowing patients with borderline molecular signatures to undergo close clinical follow-up and repeat testing if needed, while minimizing false-positive and false-negative classifications.

© 2026 Kuo H et al. *JAMA Network Open*.

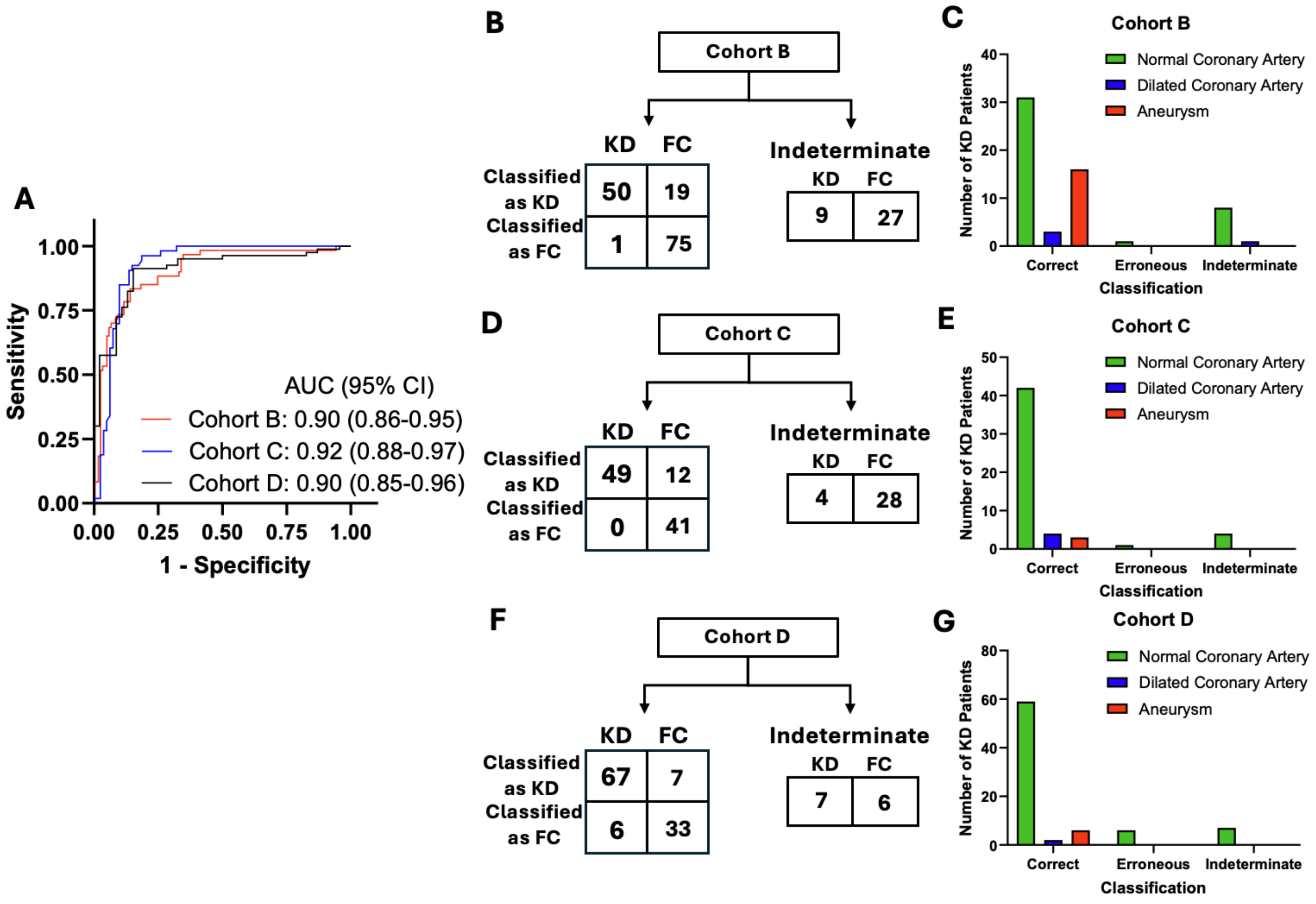

eFigure 12: KD assessment with cohort B, C, and D.

Thresholds determined from cohort A applied to cohorts B, C and D. ROC curves were generated for each cohort (A). A 2x2 table of Cohort B (B), Cohort C (D) and Cohort D (F) classification. Performance of KD score to correctly classify patients as KD with coronary artery complications in cohort B (C), cohort C (E), and cohort D (G). FCs, febrile controls; KD, Kawasaki disease.

© 2026 Kuo H et al. *JAMA Network Open*.

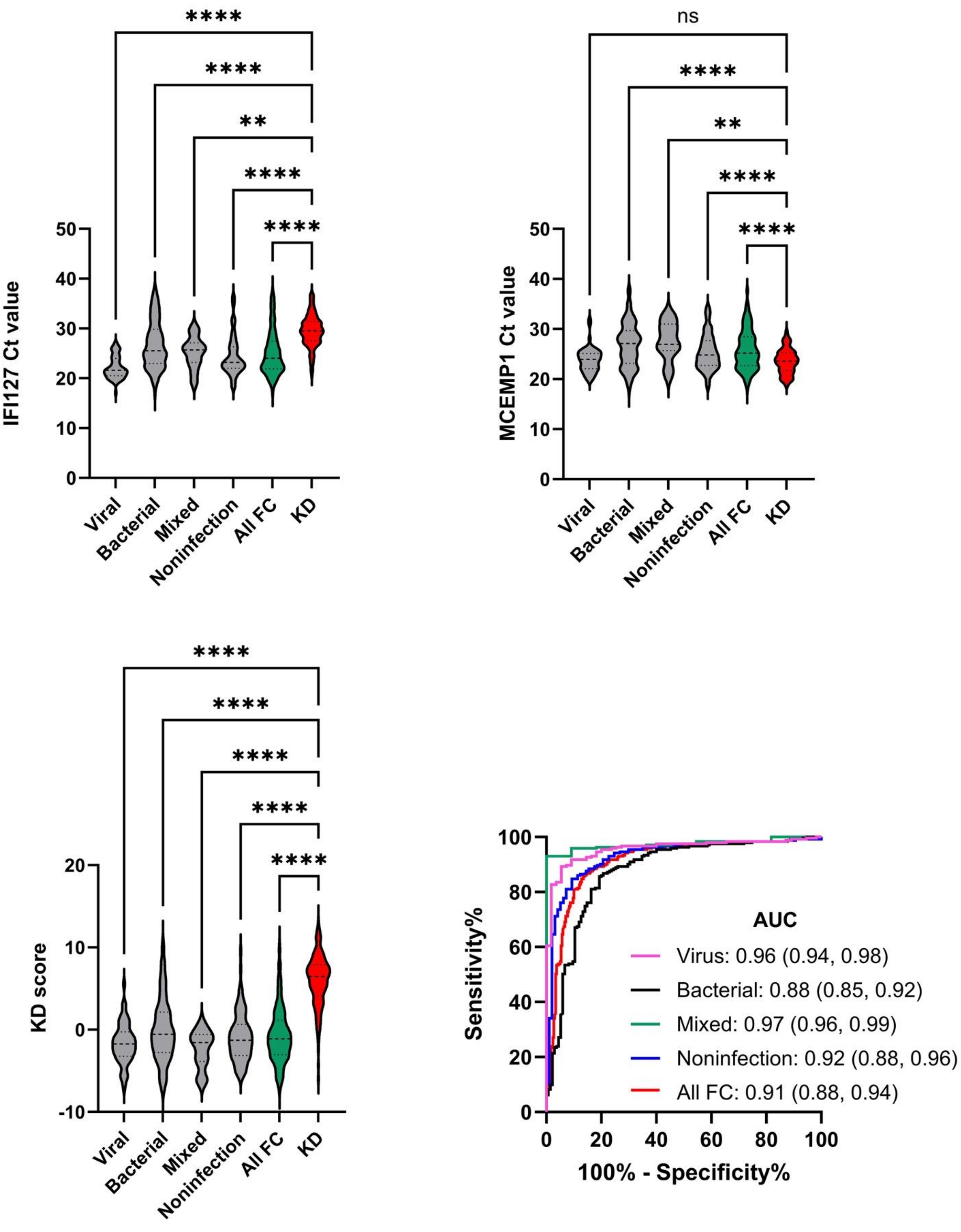

eFigure 13: KD score distinguishes pediatric febrile illness from KD (Cohort A-E). Febrile illness: viral, bacterial, mixed infections, and non-infection. * *p*<0.05, ****p*<0.0001 (Student T test) FCs, febrile controls; KD, Kawasaki disease.

© 2026 Kuo H et al. *JAMA Network Open*.

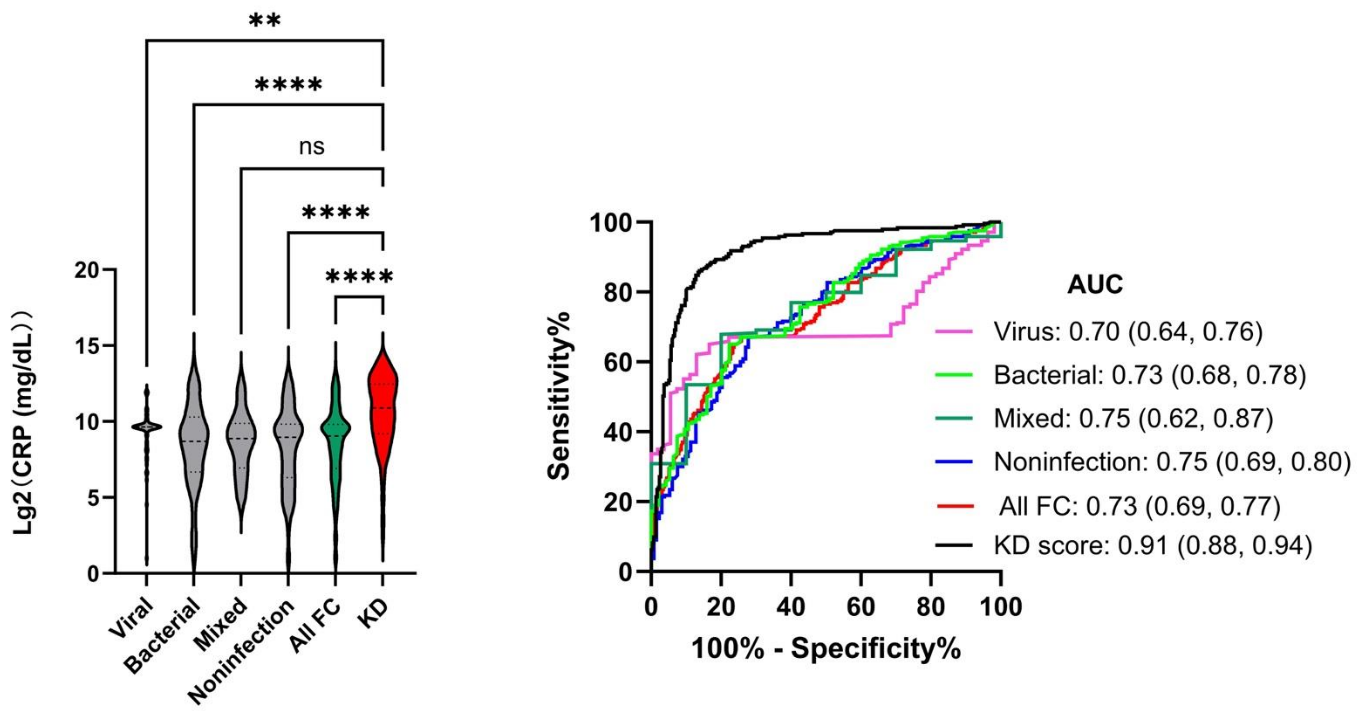

eFigure 14. Performance of C-Reactive Protein (CRP) Compared With the 2-Gene KD Score.

eFigure 14A: Distribution of CRP values across febrile control subgroups and Kawasaki disease.

Violin plots show CRP concentrations across cohorts A–E, stratified by viral, bacterial, mixed infection, and non-infection conditions. (***P < .001; ****P < .0001; Student T test). FC: febrile control; KD: Kawasaki disease.

eFigure 14B: Diagnostic performance of CRP as a comparator biomarker.

Two complementary analytic approaches were used to evaluate CRP performance:

ROC-based analysis (Youden index). Receiver operating characteristic (ROC) curves were constructed to compare the diagnostic performance of the 2-gene KD score and CRP for distinguishing KD from febrile controls.

Fixed clinical threshold analysis (CRP ⩾3 mg/dL). Across cohorts A–E, CRP values were available for 533 patients (8 missing). Using a prespecified clinical threshold of 3 mg/dL, CRP showed modest discriminatory performance for KD, with an AUC of 0.73. Sensitivity was 84.4% (205/243; 95% CI, 79.4%–88.5%), while specificity was low at 37.6% (109/290; 95% CI, 32.1%–43.1%). The negative predictive value was 74.1% (109/147), and the positive predictive value was 53.1% (205/386), reflecting a KD prevalence of 45.6% (243/533). The positive likelihood ratio was 1.35 and the negative likelihood ratio was 0.42, indicating limited standalone diagnostic utility of CRP at this clinically relevant threshold.

	Cohort A-E CRP biomarker
N	533 (Missing 8 patients)
Threshold	3 mg/dL
AUC	0.73
Sensitivity 95% CI	84.4% (205/243) 79.4% - 88.5%

© 2026 Kuo H et al. *JAMA Network Open*.

Specificity 95% CI	37.6% (109/290) 32.1% - 43.1%
NPV	74.1% (109/147)
PPV	53.1% (205/386)
Prevalence	45.6% (243/533)
LR_pos	1.352
LR_neg	0.416

© 2026 Kuo H et al. *JAMA Network Open*.

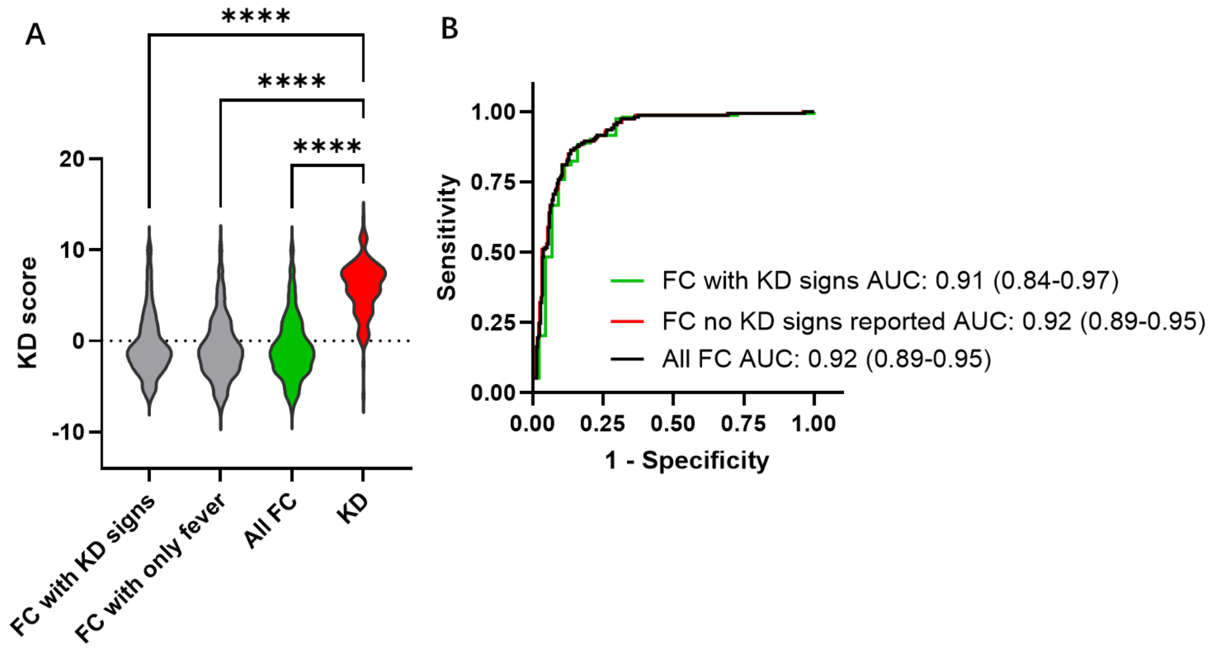

eFigure 15: The KD score separates KD patients from FC with reported KD signs.

Violin plot of the KD score of cohorts A, B, C and D separated by FC with additional KD signs (n=44), FC with only fever reported (n=244), all FC (n=288) and all KD (n=233) (a). ROC curves were generated for the KD score of the 2 genes for FC with KD signs or FC with only fever report vs KD (b). ****$p<0.0001$ (Student T test) FCs, febrile controls; KD, Kawasaki disease

© 2026 Kuo H et al. *JAMA Network Open*.

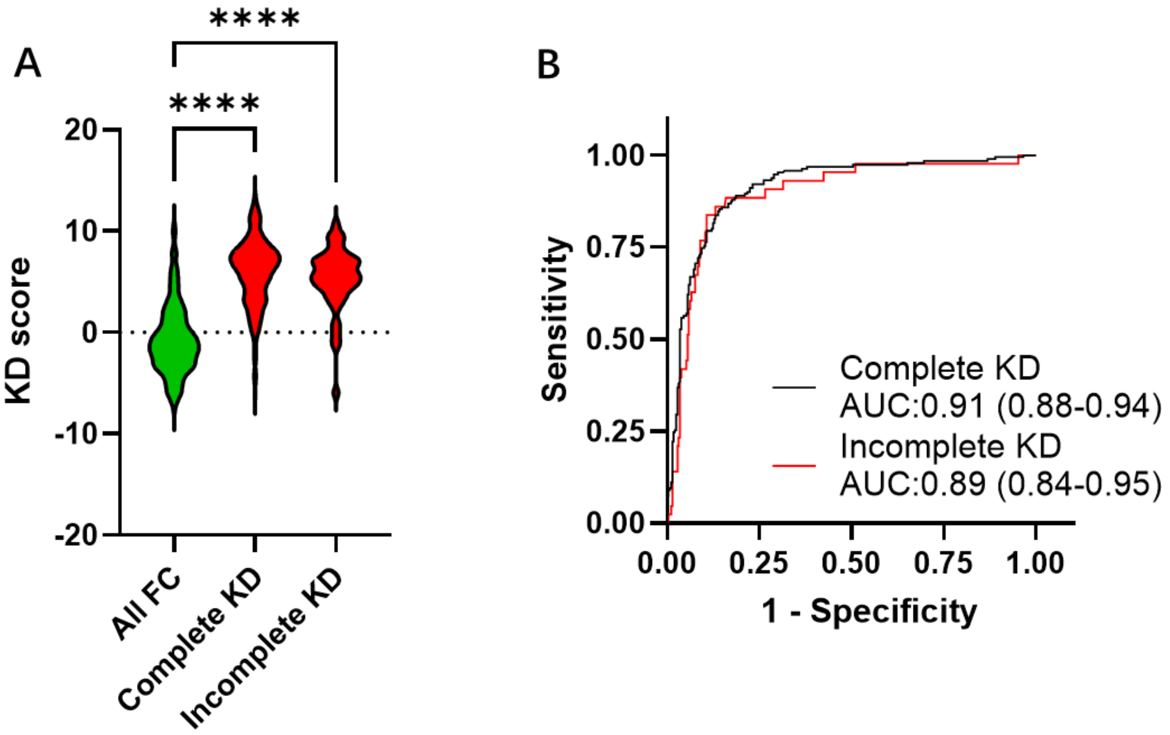

eFigure 16: The KD score identifies complete and incomplete KD.

(A) Violin plot of the KD score of cohorts A, B, C, and D separated by FC (n=288) complete KD (n=190), incomplete KD (n=43) (A) ROC curves were generated for the KD score of the 2 genes for complete KD or incomplete KD compared to FC in cohorts A, B, C, and D combined.

(B). ****$p<0.0001$ (Student T test) FCs, febrile controls; KD, Kawasaki disease.

© 2026 Kuo H et al. *JAMA Network Open*.

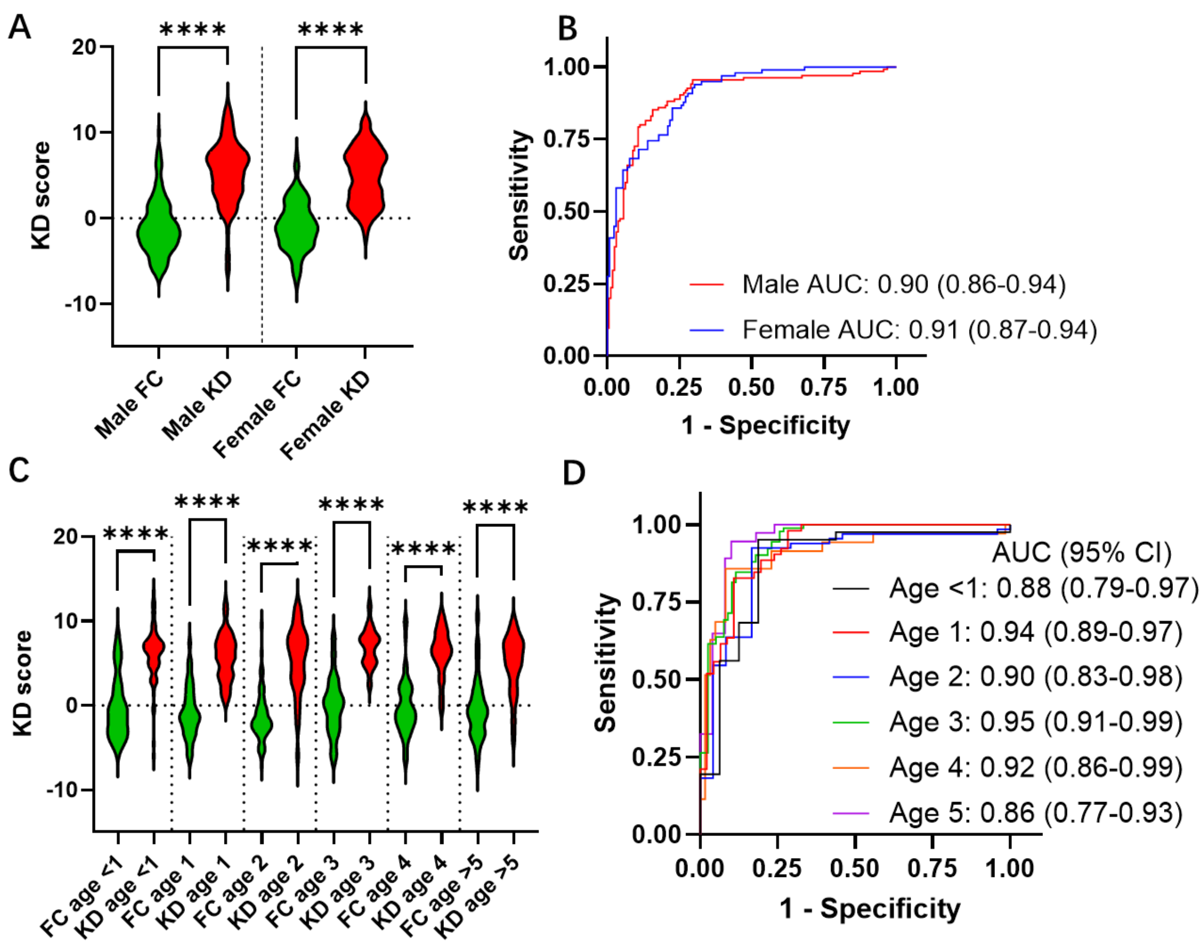

eFigure 17: The KD score performance similar cross age and sex.

Violin plot of the KD score of cohorts A, B, C and D separated by sex (A) and by age in years (C) ROC curves were generated for the KD score of the 2 genes by sex (B) and by age in years (D). Age<1 FC = 24, KD = 66; Age 1 FC=78, KD=91; Age 2 FC=61, KD=35; Age 3 FC=50 KD=37; Age 4 FC=55, KD = 23; Age >5 FC=101, KD=34. FCs, febrile controls; KD, Kawasaki disease.

© 2026 Kuo H et al. *JAMA Network Open*.

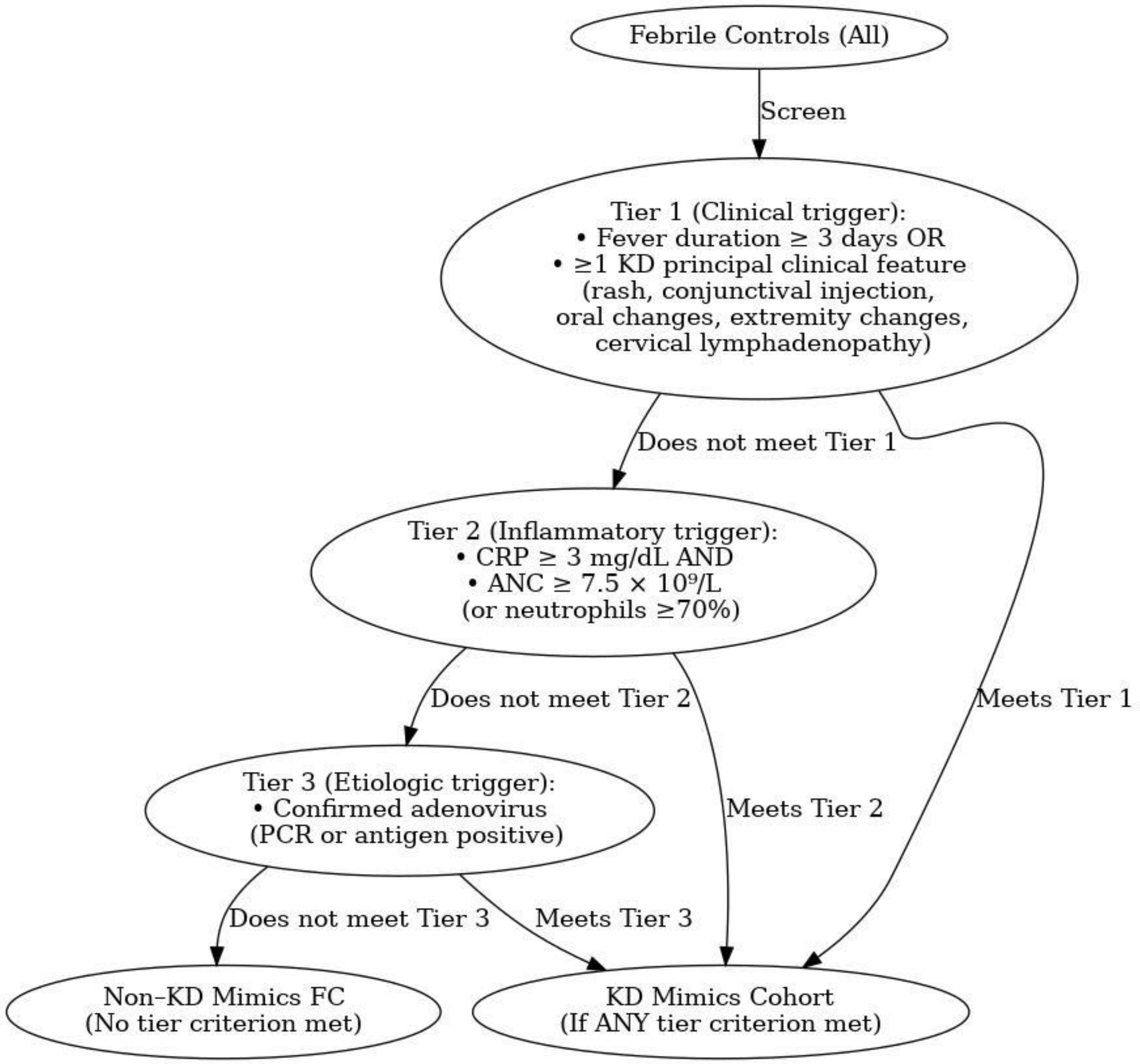

eFigure 18A: Construction of the clinically defined KD-mimic cohort.

Febrile controls were screened using a clinically motivated, multi-tier definition designed to reflect real-world evaluation for Kawasaki disease. Tier 1 identified patients in whom KD would be considered clinically, defined by fever duration of at least 3 days and the presence of at least one principal KD clinical feature. Tier 2 identified patients with a high-risk inflammatory phenotype, defined by elevated C-reactive protein (⩾3.0 mg/dL) with neutrophil predominance. Tier 3 identified patients with confirmed adenovirus infection. Among febrile controls, 8 patients had laboratory-confirmed adenovirus infection and were included in the KD-mimic comparator group. Febrile controls meeting any of these tier criteria were pooled and classified as KD mimics (n=110), and used as the comparator group for diagnostic performance analyses.

© 2026 Kuo H et al. *JAMA Network Open*.

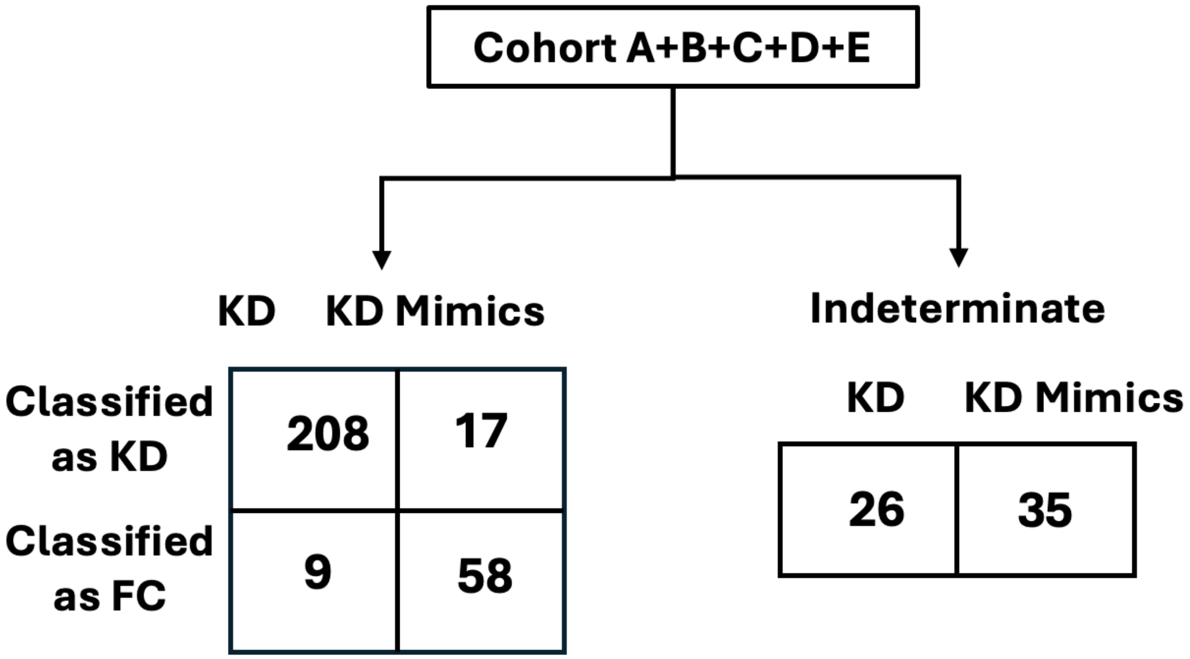

eFigure 18B: Identification of KD from clinically defined KD-mimics and performance evaluation.

When the analysis was restricted to clinically defined KD mimics, representing febrile controls in whom Kawasaki disease would plausibly be considered, the KD score continued to demonstrate strong discriminatory performance using the previously prespecified thresholds. Among patients with Kawasaki disease, 208 were correctly classified as KD and 9 were misclassified as febrile controls, while among KD mimics, 58 were correctly classified as non-KD and 17 were misclassified as KD. Importantly, a subset of patients from both groups fell into the predefined indeterminate range, including 26 KD cases and 35 KD mimics. These indeterminate results were not due to assay failure but reflect a biologically overlapping host-response state in which immediate molecular classification alone is insufficient for definitive diagnosis.

From a clinical perspective, this pattern is consistent with the assay's intended use. The test prioritizes high diagnostic certainty at the extremes of the score distribution, where most KD cases and KD mimics are correctly classified, while explicitly flagging borderline cases for closer clinical follow-up rather than forcing classification. The concentration of uncertainty within the indeterminate range reduces the risk of false-negative KD classification and minimizes inappropriate exclusion of KD in clinically ambiguous presentations. Overall, these findings demonstrate that the KD score retains clinically meaningful discrimination even under the most stringent comparator (KD mimics) and that misclassification is largely confined to biologically and clinically ambiguous cases rather than driven by fever duration or inflammatory burden alone.

© 2026 Kuo H et al. *JAMA Network Open*.

eFigure 19: KD test performance in aneurysm vs non-aneurysm KD.

In total, 243 patients with Kawasaki disease (KD) were included across all cohorts, of whom 190 had normal coronary arteries, 16 had dilated coronary arteries, and 37 had coronary artery aneurysms. Among the 37 patients with coronary artery aneurysms, 35 were correctly predicted as positive, while the remaining 2 were classified as indeterminate.

- Normal coronary artery: Z-score ≦ 2
- Dilated coronary artery: Z-score > 2 & ≦ 2.5
- Aneurysm: Z-score > 2.5

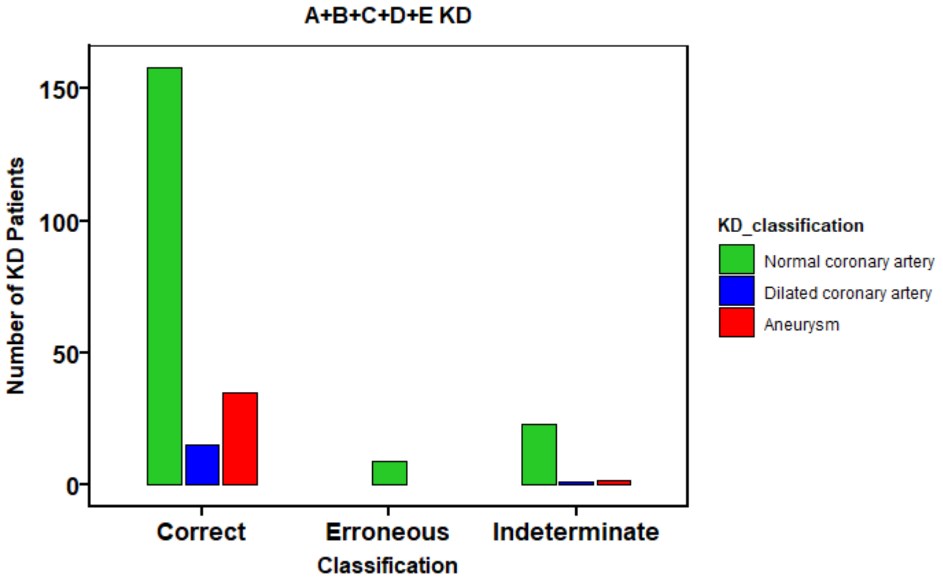

	Correct	Erroneous	Indeterminate
Normal coronary artery	158	9	23
Dilated coronary artery	15	0	1
Aneurysm	35	0	2

© 2026 Kuo H et al. *JAMA Network Open*.

Supplementary Tables

eTable 1: Kawasaki disease and vasculitis datasets included in the HMS data mining algorithm.

Dataset Name	Accession	Author	PMID	Location	Demographic	Platform	# of Kawasaki Disease samples	# of Vasculitis samples	# of Febrile Control samples
**Cohort 1: Discovery N = 204**									
	GSE15297	Popper S	19583510	USA	KD and febrile children	GPL8328	23	0	18
	GSE18606	Fury W	20600450	USA	KD and febrile children	GPL6480	20	0	9
	GSE9864	Popper S	18067656	USA	KD children	GPL6270	64	0	0
	GSE9863	Popper S	18067656	USA	KD children	GPL6271	20	0	0
	GSE33910	Okuzaki D	doi:10.4172/2155-9929.1000125	Japan	Takayasu's vasculitis adults	GPL4133	0	8	0
	GSE17114	Oliveira SA	NA	Portugal	Behcet's disease adults	GPL570	0	15	14
	GSE16945	Okuzaki D	21059707	Japan	Takayasu's vasculitis adults	GPL4133	0	13	0
**Cohort 2: Validation N = 595**									
Microarray 1	GSE73464	Wright V	35732822	UK, Spain, Netherlands & USA	KD and febrile children	GPL10558	78	0	242
Microarray 2	GSE68004	Baldwin N	29813106	USA	KD and febrile children	GPL10558	89	0	36
RNAseq 1	GSE178491	Burns J	35577777	USA	KD and febrile children	GPL20301	120	0	30
**Cohort 3: Independent Validation N = 387**									
RNAseq 2	E-MTAB-11671	Habgood-Coote D	37255317	UK, Austria, Germany, Lithuania, Spain, Switzerland, Netherlands, USA	KD and febrile children		113	0	274

© 2026 Kuo H et al. *JAMA Network Open*.

eTable 2: 13 pathways with associated genes

Pathway Name	Gene. Symbol	Gene Name
Arachidonic Acid Metabolism	ALOX5	arachidonate 5-lipoxygenase
Arachidonic Acid Metabolism	DHRS2	dehydrogenase/reductase (SDR family) member 2
Arachidonic Acid Metabolism	PLA2G4A	phospholipase A2, group IVA (cytosolic, calcium-dependent)
Arachidonic Acid Metabolism	ACSS2	acyl-CoA synthetase short-chain family member 2
Arachidonic Acid Metabolism	CYP2C8	cytochrome P450, family 2, subfamily C, polypeptide 8
Arachidonic Acid Metabolism	CYP4X1	cytochrome P450, family 4, subfamily X, polypeptide 1
CD28 Signaling in T Helper Cells	PTPRC	protein tyrosine phosphatase, receptor type, C
CD28 Signaling in T Helper Cells	ARPC1B	actin related protein 2/3 complex, subunit 1B, 41kDa
CD28 Signaling in T Helper Cells	ATM	ataxia telangiectasia mutated
CD28 Signaling in T Helper Cells	FCER1G	Fc fragment of IgE, high affinity I, receptor for; gamma polypeptide
CD28 Signaling in T Helper Cells	MALT1	mucosa associated lymphoid tissue lymphoma translocation gene 1
CD28 Signaling in T Helper Cells	HLA-DRB1	major histocompatibility complex, class II, DR beta 1
CD28 Signaling in T Helper Cells	HLA-DQA1	major histocompatibility complex, class II, DQ alpha 1
CD28 Signaling in T Helper Cells	HLA-DRA	major histocompatibility complex, class II, DR alpha
Cdc42 Signaling	ITGB5	integrin, beta 5
Cdc42 Signaling	MYL9	myosin, light chain 9, regulatory
Cdc42 Signaling	ARPC1B	actin related protein 2/3 complex, subunit 1B, 41kDa
Cdc42 Signaling	ITGAX	integrin, alpha X (complement component 3 receptor 4 subunit)
Cdc42 Signaling	RASA1	RAS p21 protein activator (GTPase activating protein) 1
Cdc42 Signaling	FCER1G	Fc fragment of IgE, high affinity I, receptor for; gamma polypeptide
Cdc42 Signaling	ITGB2	integrin, beta 2 (complement component 3 receptor 3 and 4 subunit)
Cdc42 Signaling	HLA-DRB1	major histocompatibility complex, class II, DR beta 1
Cdc42 Signaling	HLA-DQA1	major histocompatibility complex, class II, DQ alpha 1
Cdc42 Signaling	HLA-DRA	major histocompatibility complex, class II, DR alpha
Cdc42 Signaling	HLA-DRB4	major histocompatibility complex, class II, DR beta 4
Cdc42 Signaling	HLA-DPA1	major histocompatibility complex, class II, DP alpha 1
Cdc42 Signaling	HLA-B	major histocompatibility complex, class I, B
Colorectal Cancer Metastasis Signaling	PRKAR2B	protein kinase, cAMP-dependent, regulatory, type II, beta

© 2026 Kuo H et al. *JAMA Network Open*.

Colorectal Cancer Metastasis Signaling	CDH1	cadherin 1, type 1, E-cadherin (epithelial)
Colorectal Cancer Metastasis Signaling	TLR8	toll-like receptor 8
Colorectal Cancer Metastasis Signaling	AXIN1	axin 1
Colorectal Cancer Metastasis Signaling	CCND1	cyclin D1
Colorectal Cancer Metastasis Signaling	VEGFA	vascular endothelial growth factor A
Colorectal Cancer Metastasis Signaling	MMP8	matrix metallopeptidase 8 (neutrophil collagenase)
Colorectal Cancer Metastasis Signaling	GNG11	guanine nucleotide binding protein (G protein), gamma 11
Colorectal Cancer Metastasis Signaling	LEF1	lymphoid enhancer-binding factor 1
Colorectal Cancer Metastasis Signaling	TCF7L2	transcription factor 7-like 2 (T-cell specific, HMG-box)
Colorectal Cancer Metastasis Signaling	ATM	ataxia telangiectasia mutated
Colorectal Cancer Metastasis Signaling	FZD1	frizzled family receptor 1
Colorectal Cancer Metastasis Signaling	TLR7	toll-like receptor 7
Colorectal Cancer Metastasis Signaling	TNF	tumor necrosis factor
Dendritic Cell Maturation	LY75	lymphocyte antigen 75
Dendritic Cell Maturation	FSCN1	fascin homolog 1, actin-bundling protein (Strongylocentrotus purpuratus)
Dendritic Cell Maturation	COL1A1	collagen, type I, alpha 1
Dendritic Cell Maturation	CREB5	cAMP responsive element binding protein 5
Dendritic Cell Maturation	ATM	ataxia telangiectasia mutated
Dendritic Cell Maturation	CD1B	CD1b molecule
Dendritic Cell Maturation	FCER1G	Fc fragment of IgE, high affinity I, receptor for; gamma polypeptide
Dendritic Cell Maturation	FCGR3B	Fc fragment of IgG, low affinity IIIb, receptor (CD16b)
Dendritic Cell Maturation	HLA-DRB1	major histocompatibility complex, class II, DR beta 1
Dendritic Cell Maturation	HLA-DQA1	major histocompatibility complex, class II, DQ alpha 1
Dendritic Cell Maturation	FCGR1B	Fc fragment of IgG, high affinity Ib, receptor (CD64)
Dendritic Cell Maturation	HLA-DRA	major histocompatibility complex, class II, DR alpha
Dendritic Cell Maturation	HLA-DRB4	major histocompatibility complex, class II, DR beta 4
Dendritic Cell Maturation	LTB	lymphotoxin beta (TNF superfamily, member 3)
Dendritic Cell Maturation	TNF	tumor necrosis factor
Dendritic Cell Maturation	HLA-B	major histocompatibility complex, class I, B
Fc Epsilon RI Signaling	PRKCH	protein kinase C, eta
Fc Epsilon RI Signaling	PLA2G4A	phospholipase A2, group IVA (cytosolic, calcium-dependent)
Fc Epsilon RI Signaling	ATM	ataxia telangiectasia mutated
Fc Epsilon RI Signaling	MS4A2	membrane-spanning 4-domains, subfamily A, member 2
Fc Epsilon RI Signaling	FCER1G	Fc fragment of IgE, high affinity I, receptor for; gamma polypeptide
Fc Epsilon RI Signaling	JMJD7-PLA2G4B	JMJD7-PLA2G4B readthrough
Fc Epsilon RI Signaling	FCER1A	Fc fragment of IgE, high affinity I, receptor for; alpha polypeptide

© 2026 Kuo H et al. *JAMA Network Open*.

Fc Epsilon RI Signaling	TNF	tumor necrosis factor
Hepatic Fibrosis / Hepatic Stellate Cell Activation	IGF1	insulin-like growth factor 1 (somatomedin C)
Hepatic Fibrosis / Hepatic Stellate Cell Activation	IL4R	interleukin 4 receptor
Hepatic Fibrosis / Hepatic Stellate Cell Activation	MYL9	myosin, light chain 9, regulatory
Hepatic Fibrosis / Hepatic Stellate Cell Activation	COL1A1	collagen, type I, alpha 1
Hepatic Fibrosis / Hepatic Stellate Cell Activation	VEGFA	vascular endothelial growth factor A
Hepatic Fibrosis / Hepatic Stellate Cell Activation	FN1	fibronectin 1
Hepatic Fibrosis / Hepatic Stellate Cell Activation	IL1R1	interleukin 1 receptor, type I
Hepatic Fibrosis / Hepatic Stellate Cell Activation	CTGF	connective tissue growth factor
Hepatic Fibrosis / Hepatic Stellate Cell Activation	AGT	angiotensinogen (serpin peptidase inhibitor, clade A, member 8)
Hepatic Fibrosis / Hepatic Stellate Cell Activation	IGF1R	insulin-like growth factor 1 receptor
Hepatic Fibrosis / Hepatic Stellate Cell Activation	IGFBP3	insulin-like growth factor binding protein 3
Hepatic Fibrosis / Hepatic Stellate Cell Activation	TNF	tumor necrosis factor
IGF-1 Signaling	PRKAR2B	protein kinase, cAMP-dependent, regulatory, type II, beta
IGF-1 Signaling	IGF1	insulin-like growth factor 1 (somatomedin C)
IGF-1 Signaling	GRB10	growth factor receptor-bound protein 10
IGF-1 Signaling	CTGF	connective tissue growth factor
IGF-1 Signaling	IGF1R	insulin-like growth factor 1 receptor
IGF-1 Signaling	CYR61	cysteine-rich, angiogenic inducer, 61
IGF-1 Signaling	RASA1	RAS p21 protein activator (GTPase activating protein) 1
IGF-1 Signaling	IGFBP3	insulin-like growth factor binding protein 3
IGF-1 Signaling	IGFBP1	insulin-like growth factor binding protein 1
IGF-1 Signaling	ATM	ataxia telangiectasia mutated
Molecular Mechanisms of Cancer	PRKAR2B	protein kinase, cAMP-dependent, regulatory, type II, beta
Molecular Mechanisms of Cancer	PRKCH	protein kinase C, eta
Molecular Mechanisms of Cancer	GAB2	GRB2-associated binding protein 2
Molecular Mechanisms of Cancer	CDH1	cadherin 1, type 1, E-cadherin (epithelial)
Molecular Mechanisms of Cancer	ARHGEF7	Rho guanine nucleotide exchange factor (GEF) 7
Molecular Mechanisms of Cancer	AXIN1	axin 1
Molecular Mechanisms of Cancer	CCND1	cyclin D1
Molecular Mechanisms of Cancer	RAP1A	RAP1A, member of RAS oncogene family
Molecular Mechanisms of Cancer	CCND2	cyclin D2
Molecular Mechanisms of Cancer	GNG11	guanine nucleotide binding protein (G protein), gamma 11
Molecular Mechanisms of Cancer	LEF1	lymphoid enhancer-binding factor 1
Molecular Mechanisms of Cancer	RASA1	RAS p21 protein activator (GTPase activating protein) 1

© 2026 Kuo H et al. *JAMA Network Open*.

Molecular Mechanisms of Cancer	ATM	ataxia telangiectasia mutated
Molecular Mechanisms of Cancer	FZD1	frizzled family receptor 1
Molecular Mechanisms of Cancer	SIN3A	SIN3 transcription regulator homolog A (yeast)
NF-κB Signaling	TLR8	toll-like receptor 8
NF-κB Signaling	IL1R1	interleukin 1 receptor, type I
NF-κB Signaling	TNFAIP3	tumor necrosis factor, alpha-induced protein 3
NF-κB Signaling	IGF1R	insulin-like growth factor 1 receptor
NF-κB Signaling	ATM	ataxia telangiectasia mutated
NF-κB Signaling	FCER1G	Fc fragment of IgE, high affinity I, receptor for; gamma polypeptide
NF-κB Signaling	MALT1	mucosa associated lymphoid tissue lymphoma translocation gene 1
NF-κB Signaling	TLR7	toll-like receptor 7
NF-κB Signaling	TNF	tumor necrosis factor
PKCθ Signaling in T Lymphocytes	ATM	ataxia telangiectasia mutated
PKCθ Signaling in T Lymphocytes	FCER1G	Fc fragment of IgE, high affinity I, receptor for; gamma polypeptide
PKCθ Signaling in T Lymphocytes	MALT1	mucosa associated lymphoid tissue lymphoma translocation gene 1
PKCθ Signaling in T Lymphocytes	HLA-DRB1	major histocompatibility complex, class II, DR beta 1
PKCθ Signaling in T Lymphocytes	HLA-DQA1	major histocompatibility complex, class II, DQ alpha 1
PKCθ Signaling in T Lymphocytes	HLA-DRA	major histocompatibility complex, class II, DR alpha
Role of Macrophages, Fibroblasts and Endothelial Cells in Rheumatoid Arthritis	PRKCH	protein kinase C, eta
Role of Macrophages, Fibroblasts and Endothelial Cells in Rheumatoid Arthritis	TLR8	toll-like receptor 8
Role of Macrophages, Fibroblasts and Endothelial Cells in Rheumatoid Arthritis	AXIN1	axin 1
Role of Macrophages, Fibroblasts and Endothelial Cells in Rheumatoid Arthritis	SFRP1	secreted frizzled-related protein 1
Role of Macrophages, Fibroblasts and Endothelial Cells in Rheumatoid Arthritis	CCND1	cyclin D1
Role of Macrophages, Fibroblasts and Endothelial Cells in Rheumatoid Arthritis	VEGFA	vascular endothelial growth factor A
Role of Macrophages, Fibroblasts and Endothelial Cells in Rheumatoid Arthritis	FN1	fibronectin 1
Role of Macrophages, Fibroblasts and Endothelial Cells in Rheumatoid Arthritis	IL1R1	interleukin 1 receptor, type I
Role of Macrophages, Fibroblasts and Endothelial Cells in Rheumatoid Arthritis	LEF1	lymphoid enhancer-binding factor 1
Role of Macrophages, Fibroblasts and Endothelial Cells in Rheumatoid Arthritis	CREB5	cAMP responsive element binding protein 5
Role of Macrophages, Fibroblasts and Endothelial Cells in Rheumatoid Arthritis	TCF7L2	transcription factor 7-like 2 (T-cell specific, HMG-box)
Role of Macrophages, Fibroblasts and Endothelial Cells in Rheumatoid Arthritis	ATM	ataxia telangiectasia mutated
Role of Macrophages, Fibroblasts and Endothelial Cells in Rheumatoid Arthritis	FZD1	frizzled family receptor 1

© 2026 Kuo H et al. *JAMA Network Open*.

Role of Macrophages, Fibroblasts and Endothelial Cells in Rheumatoid Arthritis	F2RL1	coagulation factor II (thrombin) receptor-like 1
Role of Macrophages, Fibroblasts and Endothelial Cells in Rheumatoid Arthritis	C1S	complement component 1, s subcomponent
Role of Macrophages, Fibroblasts and Endothelial Cells in Rheumatoid Arthritis	TLR7	toll-like receptor 7
Role of Macrophages, Fibroblasts and Endothelial Cells in Rheumatoid Arthritis	LTB	lymphotoxin beta (TNF superfamily, member 3)
Role of Macrophages, Fibroblasts and Endothelial Cells in Rheumatoid Arthritis	TNF	tumor necrosis factor
Role of NFAT in Regulation of the Immune Response	MEF2BNB-MEF2B	MEF2BNB-MEF2B readthrough
Role of NFAT in Regulation of the Immune Response	KPNB1	karyopherin (importin) beta 1
Role of NFAT in Regulation of the Immune Response	GNG11	guanine nucleotide binding protein (G protein), gamma 11
Role of NFAT in Regulation of the Immune Response	ATM	ataxia telangiectasia mutated
Role of NFAT in Regulation of the Immune Response	MS4A2	membrane-spanning 4-domains, subfamily A, member 2
Role of NFAT in Regulation of the Immune Response	FCER1G	Fc fragment of IgE, high affinity I, receptor for; gamma polypeptide
Role of NFAT in Regulation of the Immune Response	FCGR3B	Fc fragment of IgG, low affinity IIIb, receptor (CD16b)
Role of NFAT in Regulation of the Immune Response	FCER1A	Fc fragment of IgE, high affinity I, receptor for; alpha polypeptide
Role of NFAT in Regulation of the Immune Response	HLA-DRB1	major histocompatibility complex, class II, DR beta 1
Role of NFAT in Regulation of the Immune Response	HLA-DQA1	major histocompatibility complex, class II, DQ alpha 1
Role of NFAT in Regulation of the Immune Response	FCGR1B	Fc fragment of IgG, high affinity Ib, receptor (CD64)
Role of NFAT in Regulation of the Immune Response	HLA-DRA	major histocompatibility complex, class II, DR alpha

© 2026 Kuo H et al. *JAMA Network Open*.

eTable 3: 54 gene candidates

Gene	Source
ABCC1	GEO
ADM	GEO
C11ORF82 (DDIAS)	Literature (Wright et al)
C1S	GEO
CACNA1E	Literature (Wright et al)
CAMK4	GEO
CASP5	Literature (Rahmati et al)
CD163	Literature (Wright et al)
CD274	GEO
CD55	GEO
CD59	GEO
CLEC4D	GEO
CLIC3	Literature (Wright et al)
CR1	GEO and literature (Rahmati et al)
CRTAM	GEO
CTGF	GEO
FCGR1B	GEO
FKBP1A	GEO
FKBP5	GEO
FKBP6	GEO
FUT7	GEO
HGF	GEO
HS.553068	Literature (Wright et al)
IFI27	Literature (Wright et al)
IFI30	GEO
KLHL2	Literature (Wright et al)
LCN2	GEO
LGALS2	GEO
LILRA5	GEO
LINC02035	Literature (Wright et al)
MAPK14	GEO
MCEMP1	GEO
MMP8	GEO
MPO	GEO
MYD88	GEO
NKTR	GEO
NOTCH4	GEO
OLFM4	GEO
PCOLCE2	GEO
PPARG	GEO
PVRL2	GEO
PYROXD2	Literature (Wright et al)
RTN1	Literature (Wright et al)
S100A12	GEO

© 2026 Kuo H et al. *JAMA Network Open*.

S100A8	GEO
S100A9	GEO
S100P	Literature (Wright et al)
SLC11A1	GEO
SLC11A2	GEO
SMOX	Literature (Wright et al)
TLR7	GEO
TREML4	GEO
VEGFA	GEO
ZNF185	Literature (Wright et al)

© 2026 Kuo H et al. *JAMA Network Open*.

eTable 4: Top 20 gene pairs

Gene Name	Meta *p* value	Meta -log10 *p* value	Log_2 Meta Fold Change	Absolute Log_2 Meta FC
IFI27_MCEMP1	**1.74E-14**	**13.76**	**-4.949918343**	**4.949918343**
CR1_IFI27	2.89E-14	13.54	4.750599989	4.750599989
IFI27_MMP8	4.23E-10	9.37	-4.580816313	4.580816313
IFI27_S100A12	8.03E-13	12.1	-4.555662574	4.555662574
IFI27_LILRA5	4.12E-14	13.39	-4.528263034	4.528263034
IFI27_SLC11A1	8.69E-14	13.06	-4.480691476	4.480691476
IFI27_S100P	1.62E-12	11.79	-4.475481349	4.475481349
CACNA1E_IFI27	5.44E-15	14.26	4.458784242	4.458784242
IFI27_KLHL2	3.24E-14	13.49	-4.457450581	4.457450581
CASP5_IFI27	4.90E-14	13.31	4.37989017	4.37989017
ADM_IFI27	7.88E-15	14.1	4.363876206	4.363876206
IFI27_PCOLCE2	4.57E-12	11.34	-4.301319943	4.301319943
IFI27_ZNF185	1.67E-14	13.78	-4.294652878	4.294652878
CD55_IFI27	3.99E-14	13.4	4.249359832	4.249359832
IFI27_MAPK14	2.68E-13	12.57	-4.17161793	4.17161793
FUT7_IFI27	1.22E-13	12.91	4.109726553	4.109726553
FCGR1B_IFI27	3.55E-14	13.45	4.066142473	4.066142473
IFI27_LCN2	1.99E-11	10.7	-4.057957417	4.057957417
IFI27_PVRL2	4.25E-13	12.37	-4.0554494	4.0554494
DDIAS IFI27	6.48E-14	13.19	4.004959425	4.004959425
IFI27_SMOX	1.01E-14	14	-3.958857489	3.958857489
FKBP1A_IFI27	1.12E-13	12.95	3.9264864	3.9264864
IFI27_PYROXD2	4.11E-15	14.39	-3.893230317	3.893230317
IFI27_MYD88	1.68E-13	12.77	-3.845039394	3.845039394
CD163_SLC11A1	3.07E-13	12.51	-1.366678615	1.366678615
CD163_KLHL2	4.77E-14	13.32	-1.27639638	1.27639638

© 2026 Kuo H et al. *JAMA Network Open*.

eTable 5: Clinical diagnoses of FC patients

Diagnoses	Cohort A	Cohort B	Cohort C	Cohort D	Cohort E	Viral	Bacterial	Mixed (viral and bacterial)
Acute Bronchiolitis	9	2				Yes	Yes	Yes
Acute Bronchitis	4	12	8			Yes	Yes	Yes
Acute Conjunctivitis	1	3			1	Yes	No	No
Acute otitis media		2				No	Yes	No
Acute Pharyngitis	2	10	1		1	Yes	Yes	Yes
Acute sinusitis	3	14			1	Yes	No	No
Acute Tonsillitis	9	28	1		3	Yes	Yes	Yes
Allergic Rhinitis	4	11			4	No	No	No
Anemia	1	2				No	No	No
Atopic Dermatitis	1	1			1	No	No	No
Bronchopneumonia	9	33	7		1	Yes	Yes	Yes
Carbuncle	1					No	Yes	No
Cellulitis		2				No	Yes	No
Convulsions	1		16			No	No	No
Croup	1	1				Yes	Yes	Yes
Dengue Fever	1	1				Yes	No	No
Enterititis	1		1			Yes	Yes	Yes
Enterobiasis		1				No	No	No
Enteroviral vesicular stomatitis		1				Yes	No	No
Erythema multiforme		1				No	No	No
Gastroduodenitis		2				No	Yes	No
Gastroenteritis or gastritis	5	9	4	7		Yes	Yes	Yes
Gingivostomatitis		1				Yes	No	No
Hand Foot and Mouth Disease		1	3			Yes	No	No
History of KD		1				No	No	No
Infectious fever			27	8		Yes	Yes	Yes
Infectious mononucleosis	1					Yes	No	No
Influenza		6		1	2	Yes	No	No
Juvenile idiopathic arthritis		1				No	No	No
Laryngopharyngitis		1				Yes	No	No
Leukopenia	1					Yes	No	No
Liver Function impairment	1					No	No	No
Lower respiratory tract infection				15		Yes	Yes	Yes
Lymphadenitis	1	3	1			Yes	Yes	Yes
Neutropenia			1			Yes	No	No
Nonthrombocytopenic purpura		1				Yes	No	No
Pneumonia	1	7	8	7	1	Yes	Yes	Yes
Septicemia		3				Yes	Yes	Yes
Upper respiratory tract infection			22	10		Yes	No	No
Urinary Tract infection	2	8	1		1	No	Yes	No
Urticaria		6			1	No	No	No
Viral Exanthem	1					Yes	No	No

© 2026 Kuo H et al. *JAMA Network Open*.

Viral Pharyngoconjunctivitis	1					Yes	No	No

© 2026 Kuo H et al. *JAMA Network Open*.

eTable 6: Clinical Characteristics for patients in the LDT validation

Characteristics	Cohort E: LDT Development (Taiwan 11/2017-3/2023)		
	FC	KD	*P* value
**# of patients**	10	10	
**Male sex, # (%)**	5 (50)	3 (30)	0.65
**Age (years), median (IQR)**	6.67 (4.05, 7.29)	2.08 (1.21, 3.46)	0.028
**Febrile day at sample collection, median (IQR)**	5 (4.2, 6.5)	6 (5,6)	0.393
**Laboratory values, median (IQR)**			
**Hemoglobin (g/dL)**	11.3 (10.93, 12.18)	11.15 (10.45, 12.23)	0.551
**CRP (mg/L)**	49.4 (31.1, 69.8)	36.3 (19.6, 58)	0.427
**Platelets (1000/uL)**	213 (171, 274.8)	329.5 (286.2, 431.5)	0.026
**WBC (1000/uL)**	9.05 (7.6, 10.45)	9.7 (8.43, 12.98)	0.253
**Mature neutrophil (%)**	57.5 (47.35, 62.6)	65.2 (50.25, 69.83)	0.728
**Immature neutrophil (%)**	0 (0, 0)	0 (0, 0)	0.368
**Absolute Neutrophil Count (ANC)**	4.42 (3.46, 6.35)	7.43 (5.03, 8.48)	0.455
**Principal KD symptoms, # (%)**			
**Lip/oral**	0 (0)	9 (90)	
**Conjunctival**	1 (10)	8 (80)	0.99
**Cervical LN**	3 (30)	3 (30)	0.07
**Extremity**	0 (0)	2 (20)	
**Rash**	4 (40)	10 (100)	
**Heart lesion status, # (%)**			
**Aneurysm**	NA	2 (20)	
**Dilated coronary artery**	NA	3 (30)	
**Normal coronary artery**	NA	5 (50)	
**Incomplete KD, # (%)**		6 (60)	
**FC diagnosis, # (%)**			
**Bacterial**	6 (60)	NA	
**Viral**	1 (10)	NA	
**Mixed infection**	0 (0)	NA	
**Noninfectious**	3 (30)	NA	

© 2026 Kuo H et al. *JAMA Network Open*.

eTable 7: Sensitivity/LLOQ of *IFI27* and *MCEMP1* assays.

RNA input		Sample 1	Sample 2	Sample 3	Sample 4	Sample 5
IFI27						
3.124 ng	Mean A	33.942	30.538	31.075	30.977	30.447
	SD A	0.098	0.058	0.040	0.156	0.054
	%CV A	0.288%	0.190%	0.128%	0.503%	0.176%
1.562 ng	Mean B	35.261	31.639	32.196	31.954	31.383
	SD B	0.311	0.180	0.213	0.079	0.124
	%CV B	0.881%	0.569%	0.661%	0.247%	0.395%
781 pg	Mean C	36.107	32.698	33.119	33.161	32.559
	SD C	0.619	0.090	0.121	0.148	0.223
	%CV C	1.715%	0.274%	0.366%	0.447%	0.686%
MCEMP1						
196 pg	Mean A	35.024	33.880	35.411	35.396	36.538
	SD A	0.268	0.321	0.495	0.289	0.455
	%CV A	0.766%	0.948%	1.397%	0.816%	1.246%
98 pg	Mean B	35.955	34.790	35.851	37.061	37.338
	SD B	0.601	0.355	0.318	0.103	0.389
	%CV B	1.673%	1.021%	0.888%	0.277%	1.043%
49 pg	Mean C	36.757	35.706	36.629	38.512	39.232
	SD C	0.310	0.159	0.754	0.660	Undetermined
	%CV C	0.842%	0.445%	2.058%	1.713%	Undetermined

Ct values of lowest linear total RNA for *IFI27* (top) and *MCEMP1* (bottom). Undetermined values and samples with unaccepeTable D or %CV do not meet required specifications and are underlined.

© 2026 Kuo H et al. *JAMA Network Open*.

eTable 8: Genomic DNA RT-qPCR results for *IFI27* and *MCEMP1*

Genomic DNA Sample	Rep1	Rep2	Rep3	Rep4	Rep5	NTC	Positive RNA (8 ng)
IFI27							
Female 100 ng	ND	ND	ND	ND	ND	ND	30.431
Female 10 ng	ND	ND	ND	ND	ND	ND	30.830
Male 100 ng	ND	ND	ND	ND	ND	ND	31.340
Male 10 ng	ND	ND	ND	ND	ND	ND	30.532
MCEMP1							
Female 100 ng	24.995	24.983	24.991	24.981	25.195	ND	27.865
Female 10 ng	28.133	28.185	28.188	28.235	28.392	ND	28.295
Male 100 ng	26.080	23.896	25.579	25.557	25.742	ND	28.157
Male 10 ng	28.900	28.816	29.095	29.175	28.912	ND	28.256

© 2026 Kuo H et al. *JAMA Network Open*.

eTable 9: Genomic DNA results with and without rDNase treatment.

Genomic DNA Sample	rDNase present?	Digestion Conditions	Ct 1	Ct 2	Ct 3	Ct 4	Ct 5	Mean Ct
Female	Yes	15 min RT	ND	ND	ND	ND	ND	NA
Male	Yes	15 min RT	31.413	31.573	31.879	32.460	32.096	31.884
Female	Yes	30 min 37C	ND	ND	ND	ND	ND	NA
Male	Yes	30 min 37C	38.720	ND	37.712	ND	ND	38.216
Female	No	15 min RT	26.547	26.498	26.497	26.834	25.700	26.415
Male	No	15 min RT	26.212	25.358	25.474	25.305	25.282	25.526
Female	No	30 min 37C	26.176	26.074	26.047	25.746	26.257	26.060
Male	No	30 min 37C	27.152	25.626	25.572	25.698	25.616	25.933

© 2026 Kuo H et al. *JAMA Network Open*.

eTable 10: Whole blood RNA results with and without rDNase treatment.

**Comparison**	**Mean Ct Difference**	**P-value (Paired T-test)**
rDNase RT vs Dnase 37C	0.023	0.722262
rDNase RT vs No Dnase RT	0.224	0.19587
rDNase 37C vs No Dnase 37C	0.020	0.869603

© 2026 Kuo H et al. *JAMA Network Open*.

eTable 11: Interfering substances testing concentrations

Interferent	Added concentration in whole blood	Pooling group
Rheumatoid Factor	45 IU/mL	**A**
**Ascorbic acid**	5.25mg/dL	**A**
**doxycycline HCl**	1.8 mg/dL	**A**
**acetylsalicylic acid**	3 mg/dL	**A**
**Human Hemoglobin**	2 g/L	**A**
**Human Serum Proteins**	60 g/L	**B**
**Triglycerides mix**	500 mg/dL	**B**
**rifampicin**	4.8 mg/dL	**B**
**ibuprofen**	21.9 mg/dL	**B**
**cefoxitin Na**	660 mg/dL	**B**
**Unconjugated Bilirubin**	20 mg/dL	**C**
**Conjugated Bilirubin**	20 mg/dL	**C**
**acetylcysteine**	15 mg/dL	**C**
**ampicillin Na**	7.5 mg/dL	**C**
**phenylbutazone**	32.1 mg/dL	**C**
**metronidazole**	12.3 mg/dL	**D**
**acetaminophen**	15.6 mg/dl	**D**
**Biotin**	0.351 mg/dL	**D**
**Creatinine**	15 mg/dL	**D**
**EDTA**	0.099 mg/dL	**D**
**Glucose**	1000 mg/dL	**E**
**Glycerol**	17 mg/dL	**E**
**Intralipid**	2000 mg/dL	**E**
**Pyruvate**	5 mg/dL	**E**
**Urea**	120 mg/dL	**E**
**Cholesterol**	400 mg/dL	**F**
**C-reactive protein (CRP)**	4 mg/dL	**F**
**soluble CD14 (sCD14) 5 µg/mL**	5 ug/mL	**F**
**Interleukin 6 (IL-6) 15 pg/mL**	15 pg/mL	**F**

© 2026 Kuo H et al. *JAMA Network Open*.

eTable 12: Interfering substances do not affect assay results

Interferent Group	Rep1 Mean	Rep2 Mean	Rep3 Mean	P-value (Paired T-test)	Difference
IFI27					
A	33.03	33.19	32.68	0.224	-0.161
B	32.82	33.63	32.84	0.781	-0.033
C	32.94	33.77	33.28	0.278	0.201
A/B/C ctrl	33.01	33.46	32.92		
D	32.26	33.29	30.97	0.246	-0.213
E	32.37	33.87	31.51	0.027	0.197
F	32.32	33.64	31.35	0.423	0.050
D/E/F ctrl	32.21	33.69	31.25		
MCEMP1					
A	30.59	30.78	31.05	0.378	-0.185
B	30.50	31.77	32.41	0.273	0.565
C	30.58	31.20	31.39	0.576	0.062
A/B/C ctrl	30.55	31.28	31.15		
D	31.01	31.34	30.10	0.914	0.021
E	31.02	31.12	30.42	0.039	0.061
F	31.05	31.22	30.29	0.535	0.058
D/E/F ctrl	30.96	31.04	30.38		

© 2026 Kuo H et al. *JAMA Network Open*.

eTable 13: Whole blood sample storage RT-qPCR results.

Sample Conditions	Rep1 Mean	Rep2 Mean	Rep3 Mean	Difference from Day 0	P-value (Paired T-test)
IFI27					
Day 0	29.27	30.98	27.77		
Day 1 4C	29.60	31.69	27.79	0.36	0.2181
Day 1 RT	29.45	32.02	28.25	0.57	0.1561
Day 2 4C	29.27	30.71	27.53	-0.17	0.1921
Day 2 RT	29.24	31.99	28.08	0.43	0.2932
Day 4 4C	28.97	31.39	28.14	0.16	0.5534
Day 4 RT	29.36	33.50	29.65	<u>1.50</u>	0.1756
Day 6 4C	29.34	32.46	27.68	0.49	0.4312
Day 6 RT	30.92	37.24	31.93	<u>4.03</u>	0.0944
MCEMP1					
Day 0	29.19	30.69	30.50		
Day 1 4C	30.32	31.26	31.38	0.86	0.0338
Day 1 RT	30.86	32.36	32.23	1.69	0.0002
Day 2 4C	29.33	30.54	30.80	0.09	0.5606
Day 2 RT	30.80	32.77	32.15	<u>1.78</u>	0.0070
Day 4 4C	29.94	31.12	32.19	0.96	0.1297
Day 4 RT	31.65	34.11	34.19	<u>3.19</u>	0.0137
Day 6 4C	30.94	32.67	31.08	1.44	0.0792
Day 6 RT	32.83	31.65	34.54	<u>2.88</u>	0.0970

Significant deviations from day 0 are underlined.

© 2026 Kuo H et al. *JAMA Network Open*.

eTable 14: Demographic/clinical characteristics and lab test results for patients with kawasaki disease and febrile controls in this study.

Note: Clinical symptoms refer to principal Kawasaki disease features, including oral mucosal changes (lip/oral), bilateral nonexudative conjunctival injection (conjunctival), cervical lymphadenopathy (cervical LN), extremity changes (extremity), and polymorphous rash (rash). ANC: absolute number of neutrophils.

Characteristics	FC	KD	P value
**# of patients**	298	243	
**Male sex, # (%)**	161 (54.4)	138 (56.8)	0.577
**Age, median (IQR)**	3.4 (1.8,5.4)	1.7 (1,3.3)	< 0.001
**Febrile day at sample collection, median (IQR)**	3 (1,5)	6 (5,7)	< 0.001
**Principal KD symptoms, # (%)**			
**Lip/oral**	5 (3.8)	228 (93.8)	< 0.001
**Conjunctival**	12 (8.7)	231 (95.1)	< 0.001
**Cervical LN**	30 (19.4)	127 (52.3)	< 0.001
**Extremity**	3 (2.3)	191 (78.9)	< 0.001
**Rash**	26 (17.8)	210 (86.4)	< 0.001
**Laboratory values, median (IQR)**			
**Hemoglobin (g/dL)**	12.1 (11.5,12.8)	10.9 (10.3,11.6)	< 0.001
**CRP (mg/L)**	52.6 (12.3,90)	190.3 (58.6,563)	< 0.001
**Platelets (1000/uL)**	247 (197.5,310.5)	361 (289,448)	< 0.001
**WBC (1000/uL)**	8 (5.9,10.8)	12.5 (10.1,16.3)	< 0.001
**Mature neutrophil (%)**	57.7 (36.3,70.6)	61.8 (47.8,71.9)	0.005
**Absolute Neutrophil Count (ANC)**	1.4 (0,3.7)	0 (0,6.7)	0.483
**Heart lesion status, # (%)**			< 0.001
**Aneurysm**		37 (15.2)	
**Dilated coronary artery**		16 (6.6)	
**Normal coronary artery**		190 (78.2)	
**Incomplete KD, # (%)**	0 (0)	49 (20.2)	< 0.001
**FC diagnosis, # (%)**			< 0.001
**Bacterial**	135 (45.3)		
**Viral**	55 (18.5)		
**Mixed infection**	11 (3.7)		
**Noninfectious**	97 (32.6)		

© 2026 Kuo H et al. *JAMA Network Open*.

eTable 15: Kawasaki disease indications in febrile controls

Cohort	A	B	C	D
Conjunctival injection	41	65	54	74
Extremity changes	31	60	35	64
Oropharyngeal changes	39	56	55	74
Skin rash	43	69	49	63
Cervical lymph node >1.5cm	24	37	40	50

eTable 16: Modeled positive and negative predictive values of the ifi27–mcemp1 assay across a range of assumed Kawasaki disease prevalences.

Positive predictive value (PPV) and negative predictive value (NPV) were modeled across a range of assumed Kawasaki disease (KD) prevalences using the observed diagnostic sensitivity (0.94) and specificity (0.82) from the primary analysis. Predictive values were calculated using Bayes' theorem according to the formulas:
PPV = (sensitivity × prevalence) ÷ [(sensitivity × prevalence) + (1 − specificity) × (1 − prevalence)] and
NPV = (specificity × [1 − prevalence]) ÷ [(specificity × [1 − prevalence]) + (1 − sensitivity) × prevalence].

Prevalence scenarios were selected to reflect clinically relevant contexts in which the assay may be applied, including very low population incidence settings (0.02%–0.08%; not intended for population screening), febrile emergency department populations (1%–3%), inpatient febrile evaluation (5%–10%), and tertiary referral populations in whom Kawasaki disease is actively suspected (20%–50%). Because predictive values depend strongly on disease prevalence, these modeled estimates provide context for interpreting diagnostic performance across different clinical settings.

Prevalence	PPV, %	NPV, %
0.02%	0.10	99.9985
0.08%	0.42	99.9941
1%	5.01	99.93
2%	9.63	99.85
3%	13.91	99.77
5%	21.56	99.62
10%	36.72	99.19
15%	47.96	98.73
20%	56.63	98.20
25%	63.51	97.62
30%	69.12	96.96
35%	73.77	96.21
40%	77.69	95.35
45%	81.03	94.35
50%	83.93	93.18

© 2026 Kuo H et al. *JAMA Network Open*.

© 2026 Kuo H et al. *JAMA Network Open*.

References

1. Akoglu H. User's guide to sample size estimation in diagnostic accuracy studies. *Turk J Emerg Med*. Oct-Dec 2022;22(4):177-185. doi:10.4103/2452-2473.357348
2. Johnson WE, Li C, Rabinovic A. Adjusting batch effects in microarray expression data using empirical Bayes methods. *Biostatistics*. Jan 2007;8(1):118-27. doi:10.1093/biostatistics/kxj037
3. Xiao Y, Hsiao TH, Suresh U, et al. A novel significance score for gene selection and ranking. *Bioinformatics*. Mar 15 2014;30(6):801-7. doi:10.1093/bioinformatics/btr671
4. Ritchie ME, Phipson B, Wu D, et al. limma powers differential expression analyses for RNA-sequencing and microarray studies. *Nucleic Acids Res*. Apr 20 2015;43(7):e47. doi:10.1093/nar/gkv007
5. Love MI, Huber W, Anders S. Moderated estimation of fold change and dispersion for RNA-seq data with DESeq2. *Genome Biol*. 2014;15(12):550. doi:10.1186/s13059-014-0550-8
6. Morgan AA, Khatri P, Jones RH, Sarwal MM, Butte AJ. Comparison of multiplex meta analysis techniques for understanding the acute rejection of solid organ transplants. *BMC Bioinformatics*. Oct 28 2010;11 Suppl 9(Suppl 9):S6. doi:10.1186/1471-2105-11-S9-S6
7. McCrindle BW, Rowley AH, Newburger JW, et al. Diagnosis, Treatment, and Long-Term Management of Kawasaki Disease: A Scientific Statement for Health Professionals From the American Heart Association. *Circulation*. Apr 25 2017;135(17):e927-e999. doi:10.1161/cir.0000000000000484
8. Burd EM. Validation of laboratory-developed molecular assays for infectious diseases. *Clin Microbiol Rev*. Jul 2010;23(3):550-76. doi:10.1128/cmr.00074-09
9. Guidelines for the Validation of Analytical Methods for Nucleic Acid Sequence-Based Analysis of Food, Feed, Cosmetics and Veterinary Products (2023).
10. Wright VJ, Herberg JA, Kaforou M, et al. Diagnosis of Kawasaki Disease Using a Minimal Whole-Blood Gene Expression Signature. *JAMA Pediatr*. Oct 1 2018;172(10):e182293. doi:10.1001/jamapediatrics.2018.2293
11. Rahmati Y, Mollanoori H, Najafi S, Esmaeili S, Alivand MR. CASP5 and CR1 as potential biomarkers for Kawasaki disease: an Integrated Bioinformatics-Experimental Study. *BMC Pediatr*. Dec 11 2021;21(1):566. doi:10.1186/s12887-021-03003-5

© 2026 Kuo H et al. *JAMA Network Open*.
