# Supplementary material for: Validation of a 2-Gene Blood Test for Kawasaki Disease in Febrile Children
Source: JAMA Netw Open. 2026 May 28;9(5):e2615420. doi: 10.1001/jamanetworkopen.2026.15420 (PMC13220111; doi:10.1001/jamanetworkopen.2026.15420)
Supplement: Supplement 2. — Data Sharing Statement [file jamanetwopen-e2615420-s002.pdf]

Data Sharing Statement

Kuo. Validation of a 2-Gene Blood Test for Kawasaki Disease in Febrile Children. *JAMA Netw Open*. Published May 28, 2026. doi:10.1001/jamanetworkopen.2026.15420

Data
Data available: Yes
Data types: Deidentified participant data
How to access data: This study utilized electronic health records from patients at Chang Gung Memorial Hospital in Taiwan and the Children's Hospital of Fudan University in Shanghai, who provided consent for research use by the respective institutions. To protect patient privacy and comply with institutional ethical approvals, individual-level clinical data are not publicly available. De-identified summary data, analytic code, and materials necessary to reproduce the reported analyses will be available at https://github.com/bxlinglaboratory/Diagnostic-gene-signatures-for-Kawasaki-disease upon publication. Access to additional de-identified data may be considered through collaboration with Chang Gung Memorial Hospital and Children's Hospital of Fudan University, subject to institutional review and approval.
When available: With publication

Supporting Documents
Document types: Statistical/analytic code
How to access documents: https://github.com/bxlinglaboratory/Diagnostic-gene-signatures-for-Kawasaki-disease
When available: With publication

Additional Information
Who can access the data: Any academic or industrial researcher.
Types of analyses: This study utilized electronic health records from patients at Chang Gung Memorial Hospital in Taiwan and the Children's Hospital of Fudan University in Shanghai, who consented to have their data used for research purposes by the hospital's investigators. To protect patient privacy and comply with the original ethical approval, the dataset cannot be made publicly available without obtaining additional consent or ethical clearance, as this could compromise confidentiality. De-identified participant data supporting the reported findings will be available at https://github.com/bxlinglaboratory/Diagnostic-gene-signatures-for-Kawasaki-disease upon publication of this work. Any further analyses will be conducted in collaboration with Chang Gung Memorial Hospital and Children's Hospital of Fudan University.
Mechanisms of data availability: Any further analyses will be conducted in collaboration with Chang Gung Memorial Hospital and Children's Hospital of Fudan University.
Any additional restrictions: N/A
